# Direct visualization of single-molecule membrane protein interactions in living cells

**DOI:** 10.1371/journal.pbio.2006660

**Published:** 2018-12-13

**Authors:** Do-Hyeon Kim, Soyeon Park, Dong-Kyun Kim, Min Gyu Jeong, Jungeun Noh, Yonghoon Kwon, Kai Zhou, Nam Ki Lee, Sung Ho Ryu

**Affiliations:** 1 Department of Life Sciences, Pohang University of Science and Technology, Pohang, Republic of Korea; 2 School of Interdisciplinary Bioscience and Bioengineering, Pohang University of Science and Technology, Pohang, Republic of Korea; 3 Integrative Biosciences and Biotechnology, Pohang University of Science and Technology, Pohang, Republic of Korea; 4 Department of Chemistry, Seoul National University, Seoul, Republic of Korea; University of California, Berkeley, United States of America

## Abstract

Interactions between membrane proteins are poorly understood despite their importance in cell signaling and drug development. Here, we present a co-immunoimmobilization assay (Co-II) enabling the direct observation of membrane protein interactions in single living cells that overcomes the limitations of currently prevalent proximity-based indirect methods. Using Co-II, we investigated the transient homodimerizations of epidermal growth factor receptor (EGFR) and beta-2 adrenergic receptor (β2-AR) in living cells, revealing the differential regulation of these receptors’ dimerizations by molecular conformations and microenvironment in a plasma membrane. Co-II should provide a simple, rapid, and robust platform for visualizing both weak and strong protein interactions in the plasma membrane of living cells.

## Author summary

Protein–protein interactions govern cellular processes. The majority of these physical interactions previously identified are strong/permanent interactions, which typically remain unbroken even after purification. The weak/transient interactions between proteins have been implicated in the control of dynamic cellular process that maintain cellular homeostasis and trigger signaling cascades upon environmental changes. However, these interactions are poorly investigated, mainly due to the methodological limitations. Here, we have developed a co-immunoimmobilization assay called Co-II that enables the direct visualization of protein–protein interactions in the membrane of living cells at the single-molecule level. Co-II is based on the intuitive concept that if the protein of interest is immobilized, the interacting protein must be co-immobilized. The use of intrinsic protein diffusivity fundamentally overcomes the limitations of proximity-based methods. Using Co-II, we study the transient homodimerizations of EGFR and β2-AR in living cells, which have been implicated in several types of cancers and heart diseases. We show that the dimerization of these receptors is differently regulated by molecular conformations and the microenvironment in the plasma membrane.

## Introduction

Membrane proteins play crucial roles in communication between intracellular and extracellular environments across cell membranes [1]. Malfunctioning of membrane proteins often results in myriad diseases [2], which makes these proteins major therapeutic targets [3]. Despite their importance in cell signaling and drug development, however, membrane protein interactions in living cells have been poorly understood due to methodological limitations [4].

Various methods to investigate membrane protein interactions have been developed over several decades, such as chemical cross-linking, yeast two-hybrid (Y2H), and fluorescence resonance energy transfer (FRET) [5,6]. Nevertheless, the intrinsic principles of these assays are actually the same: proximity between a bait protein (protein of interest) and a prey protein (binding partner) is utilized for the measurement of their interaction. The use of proximity between two proteins as an indirect indicator for their physical interaction can produce false positives, especially when the interactions in a crowded membrane are investigated [7]. Furthermore, the readout signals of these assays rely on the distance between the tags of a bait and a prey, which varies the results depending on the tag orientation on the proteins and makes it difficult to directly and quantitatively translate the result into the strength of the interaction [8,9].

The dimerization of receptors in a plasma membrane is a critical process for receptor activation [10]. Although the structural aspect of receptor dimerization has been intensively studied [11,12], information about the dynamics of the dimerization in a plasma membrane still remains elusive. The characterization of transient dimerization under various conditions such as drug treatment or mutations is particularly difficult, mainly due to the limited ability of current tools to capture the rapid moment of the dynamic interaction in the crowded membrane of living cells [4,5,13,14].

Here, we established an in situ imaging method that directly captures the membrane protein interactions in living cells on the basis of the protein’s inherent diffusivity by utilizing the synergy between single-particle tracking (SPT) and antibody-induced protein immobilization, of which powerfulness to assess the protein–protein interaction was previously demonstrated [15]. The interaction between prey and bait proteins was visualized through the co-immobilization (Co-II) of the prey with the immobilized bait. Then, the co-immobilizing event was counted at the single-molecule level using single-particle tracking photoactivated localization microscopy (sptPALM) [16], allowing us to determine and compare the strength of the interactions in the membrane of living cells. Using Co-II, we revealed that epidermal growth factor receptor (EGFR) and beta-2 adrenergic receptor (β2-AR) homodimerization are dominantly regulated by the intramolecular conformation and membrane microenvironment, respectively.

## Results

### Membrane protein interactions are directly visualized using co-immunoimmobilization (Co-II)

**Fig 1:**
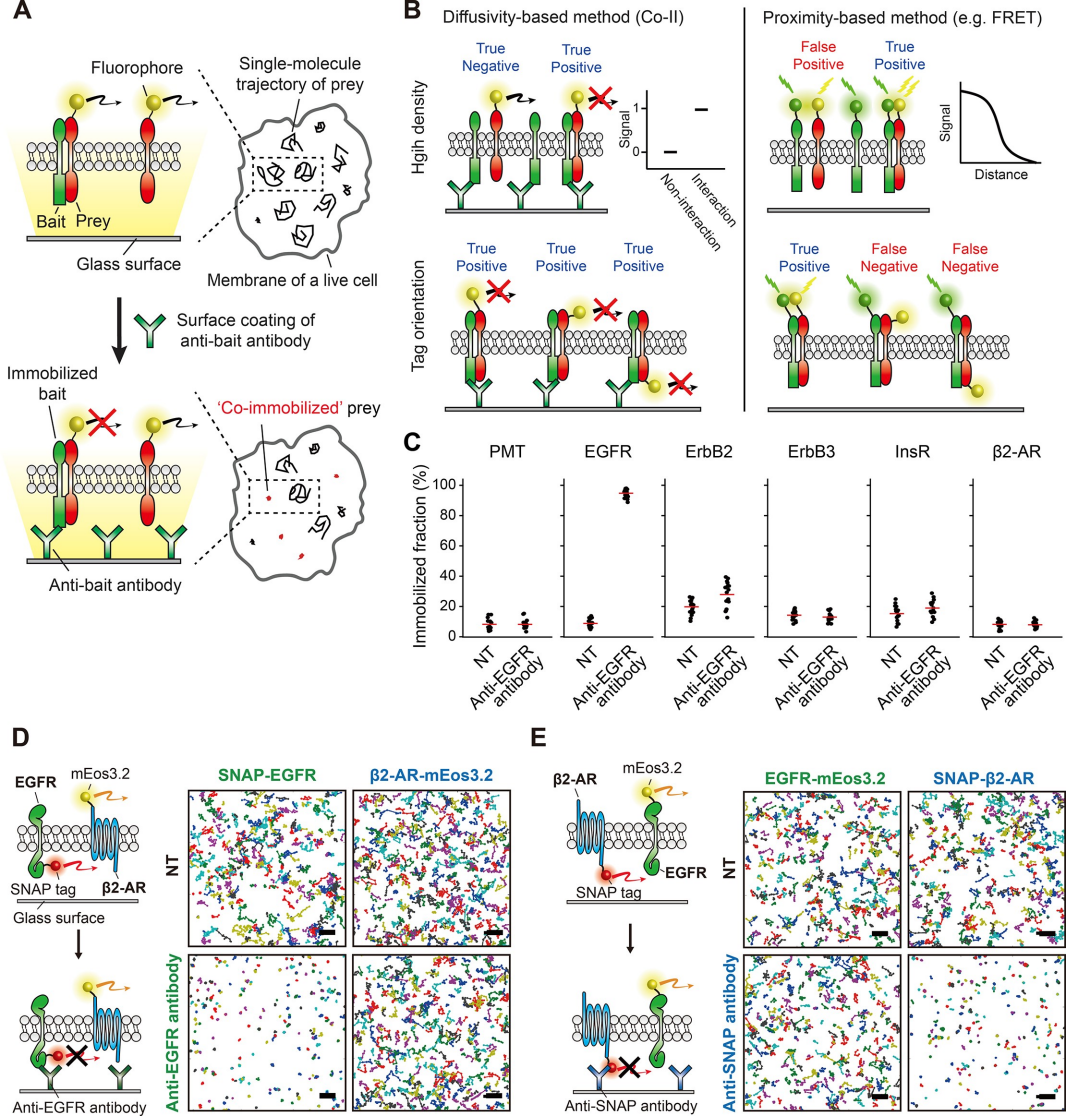
Membrane protein interactions are directly visualized using co-immunoimmobilization (Co-II). (A) Schematic of the Co-II assay. The interaction between a fluorescently labeled prey protein and a bait protein is specifically probed by the co-immobilized prey produced after antibody-induced immobilization of the bait protein, which is visualized using sptPALM in single living cells. (B) Comparison between a diffusivity-based method (Co-II) and a proximity-based method (e.g., FRET). In the crowded membrane of living cells, Co-II specifically detects genuine interactions between membrane proteins, while the proximity-based methods are vulnerable to producing false positive signals because a prey and a bait are located nearby. Co-II captures membrane protein interactions independent of tag orientation, while the proximity-based methods require a careful design for donor–acceptor orientation. (C) The bait-specific immobilization using a surface-coated antibody in living cells. The immobilized fractions of PMT, EGFR, ErbB2, ErbB3, InsR, and β2-AR in multiple cells before (NT) and after anti-EGFR antibody treatment. Examined membrane proteins were expressed at a level at least 10 times higher than the expression level of EGFR to avoid the specific co-immobilization resulting from the genuine interaction with EGFR. Each dot represents single-cell data, and the red solid lines indicate the average of the immobilized fraction obtained from multiple cells (*n* > 10). (D–E) Illustration and trajectory maps for validation of molecule-specific immobilization in the plasma membrane of a living cell. A total of 400 trajectories are shown in each trajectory map. Scale bar, 2 μm. SNAP-EGFR was specifically and almost completely immobilized by anti-EGFR antibody treatment, whereas the immobilized fraction of β2-AR-mEos3.2 was not altered (D). Specific immobilization of β2-AR against EGFR was confirmed vice versa using SNAP-β2-AR and EGFR-mEos3.2 with anti-SNAP antibody (E). β2-AR, beta-2 adrenergic receptor; EGFR, epidermal growth factor receptor; ErbB2, erb-b2 receptor tyrosine kinase 2; ErbB3, erb-b2 receptor tyrosine kinase 3; FRET, fluorescence resonance energy transfer; InsR, insulin receptor; mEos3.2, monomeric Eos fluorescent protein variant 3.2; NT, not treated; PMT, plasma membrane targeting; SNAP, SNAP-tag; sptPALM, single-particle tracking photoactivated localization microscopy.

To directly visualize protein–protein interactions in the plasma membrane of living cells at the single-molecule level, a bait protein (a protein of interest) on a cell membrane is specifically immobilized using its antibody coated on a glass surface. Then, a prey protein (an interacting partner) that diffuses on the plasma membrane is immobilized together with the bait protein whenever the interaction occurs, which provides a direct indicator of their physical interactions (Fig 1A). This co-immobilized moment of the prey protein with the bait protein is captured by sptPALM [16]. By counting the number of co-immobilized single-molecule trajectories specifically generated by the prey–bait interaction after the immobilization of the bait protein, the strength of the interactions can be quantitatively determined, allowing linear comparisons between the two interactions. We call this method co-immunoimmobilization (Co-II). Co-II overcomes the limitations derived from the use of proximity, including false positives at high density, dependency on tag orientation, and difficulty of quantification (Fig 1B).

Complete immobilization of bait proteins is critical for Co-II implementation; otherwise, the interactions between the prey and bait proteins do not always produce co-immobilized trajectories. We examined the efficiency of the immunoimmobilization using EGFR. To build an antibody-coated coverslip, we prepared a thiol-functionalized coverslip using 3-mercaptopropyl-trimethoxysilane. Next, we utilized maleimide-activated neutravidin to covalently passivate the neutravidin to the coverslip and then added the biotin-conjugated antibody. Using COS7 cells transiently expressing EGFR tagged with monomeric Eos fluorescent protein variant 3.2 (mEos3.2) at its C terminus (EGFR-mEos3.2), we analyzed the immobilized fraction of EGFR, which increased after the addition of the anti-EGFR antibody using sptPALM. To quantify the amount of the immobilized fraction, we calculated short-time diffusion coefficients from the trajectories to define immobilization in terms of diffusivity using mean squared displacement (MSD) = 4DΔt + 4e^2^ (0 < Δt < 780 ms). The diffusion coefficient criteria for classifying immobilization were determined based on a localization error. Nearly complete EGFR immobilization (>93.3%) was achieved when the secondary antibody was adopted between the neutravidin and anti-EGFR antibody to adjust the height between a glass surface and a plasma membrane (S1 Fig). The immobilization efficiency was independent of the expression level or the binding epitopes of EGFR targeted by different antibodies (S1 Fig). The even immobilization of the bait proteins was achieved across the entire cell surface within 15 min at 100 μg/mL of the antibody (S2 Fig). Another major concern for Co-II implementation was whether the immobilization of EGFR is specific to all the membrane proteins coexisting in a plasma membrane; otherwise, the co-immobilization of a prey protein would result from the interaction with nonspecifically immobilized proteins, not only with the intended bait protein. We verified that the immobilization of EGFR using the anti-EGFR antibody coated on a glass surface did not immobilize various membrane proteins, including erb-b2 receptor tyrosine kinase 2 (ErbB2), erb-b2 receptor tyrosine kinase 3 (ErbB3), insulin receptor, β2-AR, and plasma membrane targeting (PMT) signal peptide, which force mEos3.2 to localize on a plasma membrane. (Fig 1C). The immobilization of EGFR did not alter the spatial organization of EGFR distribution on the plasma membrane (S2 Fig). Furthermore, no cross-linking of the bait proteins induced by the anti-bait antibody was observed, as the excess amount of the antibody compared with the bait protein was coated on the glass surface (S2 Fig).

To further evaluate the specificity of the immunoimmobilization, we simultaneously monitored the EGFR and β2-AR trajectories in a single cell using mEos3.2 and a SNAP tag labeled with benzyl-guanine–conjugated CF660R, respectively, before and after the addition of the anti-EGFR antibody (Fig 1D). When EGFR was immobilized with 98.1% of an immobilized fraction, the immobilized β2-AR fraction was not altered significantly (S3 Fig). This specificity of the immobilization between EGFR and β2-AR was confirmed vice versa (Fig 1E and S3 Fig). These results showed that Co-II can be simply and robustly implemented and should provide a direct indicator of the protein–protein interactions in the plasma membrane of living cells.

### Equilibrium dissociation constant of EGFR pre-dimerization is determined using Co-II

**Fig 2:**
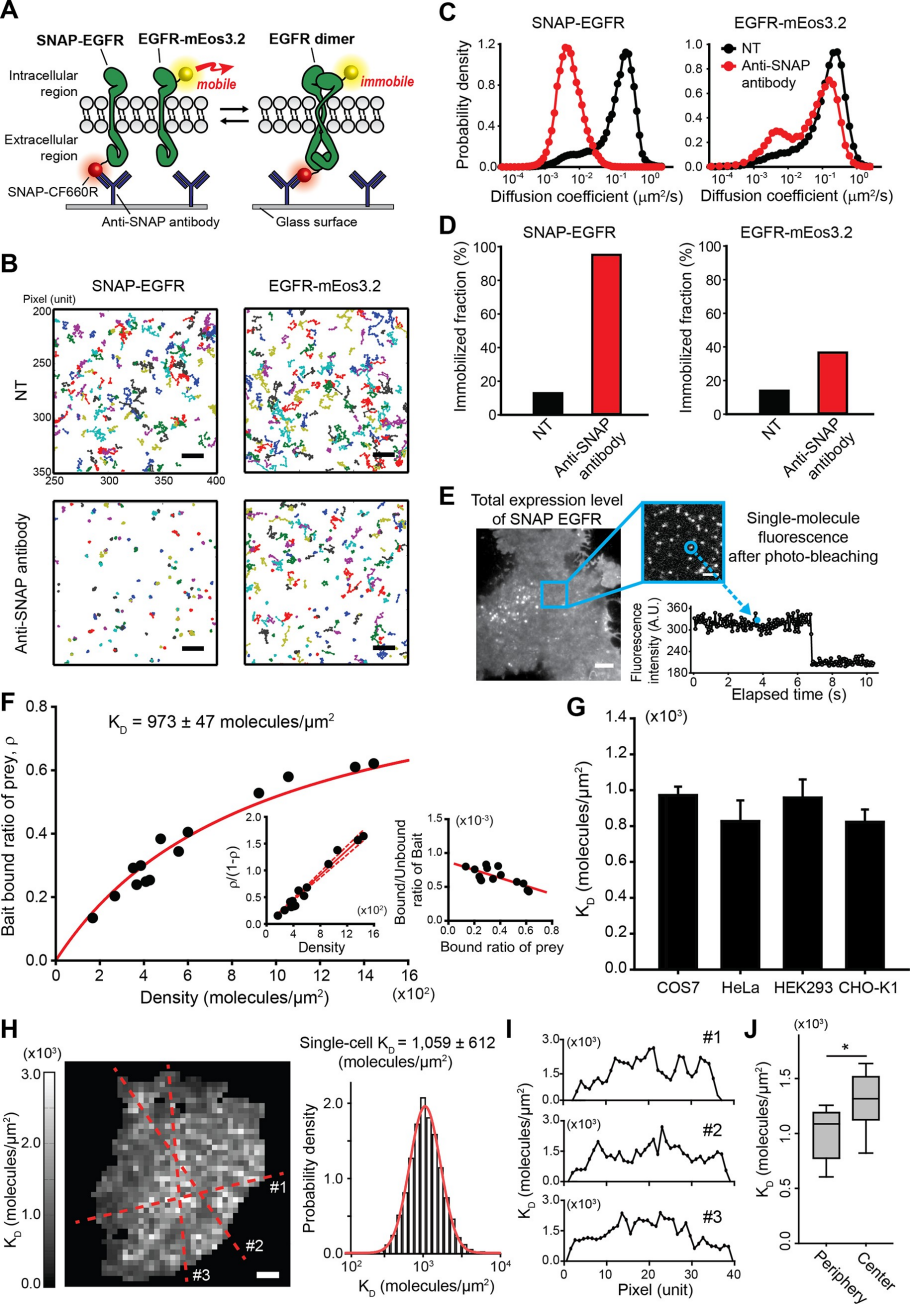
Equilibrium dissociation constant of EGFR pre-dimerization is determined using Co-II. (A) Schematic representation of the K_D_ measurement of EGFR homodimerization using Co-II. EGFR-mEos3.2 becomes co-immobilized only when interacting with the surface-immobilized SNAP-EGFR by an anti-SNAP antibody; otherwise, it remains in a mobile state. (B) Trajectory map of CF660R-labeled SNAP-EGFR and EGFR-mEos3.2 before and after anti-SNAP antibody treatment in the same single COS7 cell growing with 10% FBS. A total of 200 trajectories are shown in each trajectory map. Scale bar, 3 μm. (C) Diffusion-coefficient distribution of SNAP-EGFR and EGFR-mEos3.2 before (black line) and after anti-SNAP antibody treatment (red line). The immobilization criteria are presented as a blue dashed line. (D) The immobilized fractions of SNAP-EGFR and EGFR-mEos3.2 before and after anti-SNAP antibody treatment. (E) Fluorescence images of total expression and single-molecule–level expression of SNAP-EGFR. Scale bars, 5 μm and 2 μm, respectively. A fluorescence intensity profile of single SNAP-EGFR shows a single bleaching step. (F) K_D_ analysis using a binding curve of prey EGFR to bait EGFR (y-axis) with respect to the density of the antibody-induced immobilized bait EGFR (x-axis). The bound/unbound ratio of the prey with respect to the density is shown (left inset) with a linear fit (red solid line) and a 95% confidence interval (red dashed lines). Scatchard plot for EGFR pre-homodimerization is shown (right inset). The K_D_ was determined in DMEM supplemented with 10% FBS at 37 °C. Each dot indicates data obtained from individual cells. (G) K_D_ of EGFR pre-homodimerization measured in various cell lines. The error bars represent the SEM at the single-cell level (*n* > 4). (H) A spatial K_D_ map of EGFR pre-homodimerization and the log-normal distribution of the K_D_ values obtained from different regions of plasma membrane in a single living cell. Scale bar, 5 μm. (I) The K_D_ profiles obtained from the cross sections corresponding to the red dashed lines in panel H. (J) The box plots displaying the distributions of K_D_ values obtained from periphery or center regions of each single cell. *n* = 10. *p < 0.05 (Student *t* test). A.U., arbitrary unit; DMEM, Dulbecco's Modified Eagle Medium; EGFR, epidermal growth factor receptor; FBS, fetal bovine serum; mEos3.2, monomeric Eos fluorescent protein variant 3.2; SNAP, SNAP-tag.

Using Co-II, we quantitatively measured EGFR pre-homodimerization (ligand-independent dimerization) in a live COS7 cell by utilizing EGFR-mEos3.2 as a prey and SNAP-EGFR as a bait (Fig 2A). To minimize the dimerization events between two mobile EGFR-mEos3.2 proteins, we excessively expressed SNAP-EGFR compared with EGFR-mEos3.2, which allows us to assume the dimerization process as a pseudo-first-order reaction for the determination of an equilibrium dissociation constant, K_D_ (See the detail in Materials and methods). We tracked CF660R-labeled SNAP-EGFR and EGFR-mEos3.2 before and after the addition of the anti-SNAP antibody (Fig 2B). The mobile subpopulation of SNAP-EGFR was almost fully shifted into the immobile subpopulation (95.2%), whereas only a partial shift (22.7%) was observed for EGFR-mEos3.2 (Fig 2C and 2D), which represents the amount of the physical interaction between the mobile EGFR-mEos3.2 and the immobilized SNAP-EGFR at dynamic equilibrium. We also observed the transient colocalization of the prey EGFR with the immobilized bait EGFR at the single-molecule level, which supports that the co-immobilization of the prey EGFR is derived from a physically interacting process (S4 Fig).

Next, we determined the concentration of the immobilized bait EGFR in the plasma membrane, which is determined by the concentration of SNAP-EGFR multiplied by the anti-SNAP antibody-induced immobilization fraction of SNAP-EGFR. The concentration of SNAP-EGFR on the COS7 cell surface was measured by normalizing the total fluorescence intensity by the single-molecule intensity [17–19] (Fig 2E). We obtained a total internal reflection fluorescence (TIRF) image for CF660R-labeled SNAP-EGFR prior to the tracking procedure. After the tracking procedures were finished, we acquired TIRF images for single-molecule SNAP-EGFRs by photobleaching until individual SNAP proteins were spatially resolved. We collected single-molecule SNAP-EGFRs that exhibited a one-step photobleaching trace to calculate the average fluorescent intensity emitted from a single CF660R dye. We additionally corrected the total number of SNAP-EGFRs, considering the proportion of nonfluorescent CF660R, which should be immobilized but not detected [20] (S5 Fig). Because membrane proteins diffuse laterally on a two-dimensional plasma membrane, we used a density notation instead of molar concentration because the definition of molarity in a plasma membrane is currently ambiguous [18,21,22]. We assumed that the plasma membrane is flat because the in situ measurement of the actual geometry of the dynamic plasma membrane is technically currently limited [18], which may cause bias in the estimation of the concentration [21].

We analyzed the dependency of the co-immobilized fraction of EGFR-mEos3.2 with respect to the expression level of SNAP-EGFR (Fig 2F). The K_D_ of EGFR pre-homodimerization in the single cell was 973 ± 47 molecules/μm^2^ (mean ± SEM) in Dulbecco's Modified Eagle Medium (DMEM) supplemented with 10% fetal bovine serum (FBS) at 37 °C. Analysis using a Scatchard plot confirmed that Co-II measured the pseudo-first-order reaction of the EGFR pre-dimerization (Fig 2F). This result indicates that the major portion of the interaction between the mobile prey and the immobilized bait is a dimerization process at the single-cell level in the concentration range we measured. The K_D_ values of the EGFR pre-dimerization in various cell lines, including HeLa, HEK293, and CHO-K1 cells, did not vary significantly (Fig 2G), indicating that the contexts of plasma membrane from these cell lines marginally affect the EGFR pre-dimerization. No photodamage was detected in the cells after the Co-II assay (S6 Fig).

The in situ capability of Co-II allows us to acquire a spatial K_D_ map in a single living cell. The power of sptPALM to obtain the sufficient number of trajectories in a single cell enables us to determine the K_D_ in a small area of the single cell. We constructed a K_D_ map of EGFR pre-homodimerization in a single living cell at a 1.2-μm resolution (Fig 2H). The distribution of the K_D_ values obtained from different regions of plasma membrane showed a log-normal distribution with a K_D_ of 1,059 ± 612 molecules/μm^2^ (mean ± SD). We found a geometric tendency of K_D_ values in the plasma membrane to be lower at the periphery and higher at the center (Fig 2I and 2J and S7 Fig), which is consistent with the previous report regarding the spatial control of EGFR activation [23]. Although this spatial heterogeneity of K_D_ values might result from the bias in receptor concentration derived from the assumption of a flat membrane, this spatial heterogeneity implies that the intrinsic characteristics of EGFR pre-homodimerization might be controlled by the cellular microenvironment in living cells.

### EGFR dimerization is distinctively regulated by various inter- and intramolecular perturbations in the membrane of living cells

**Fig 3:**
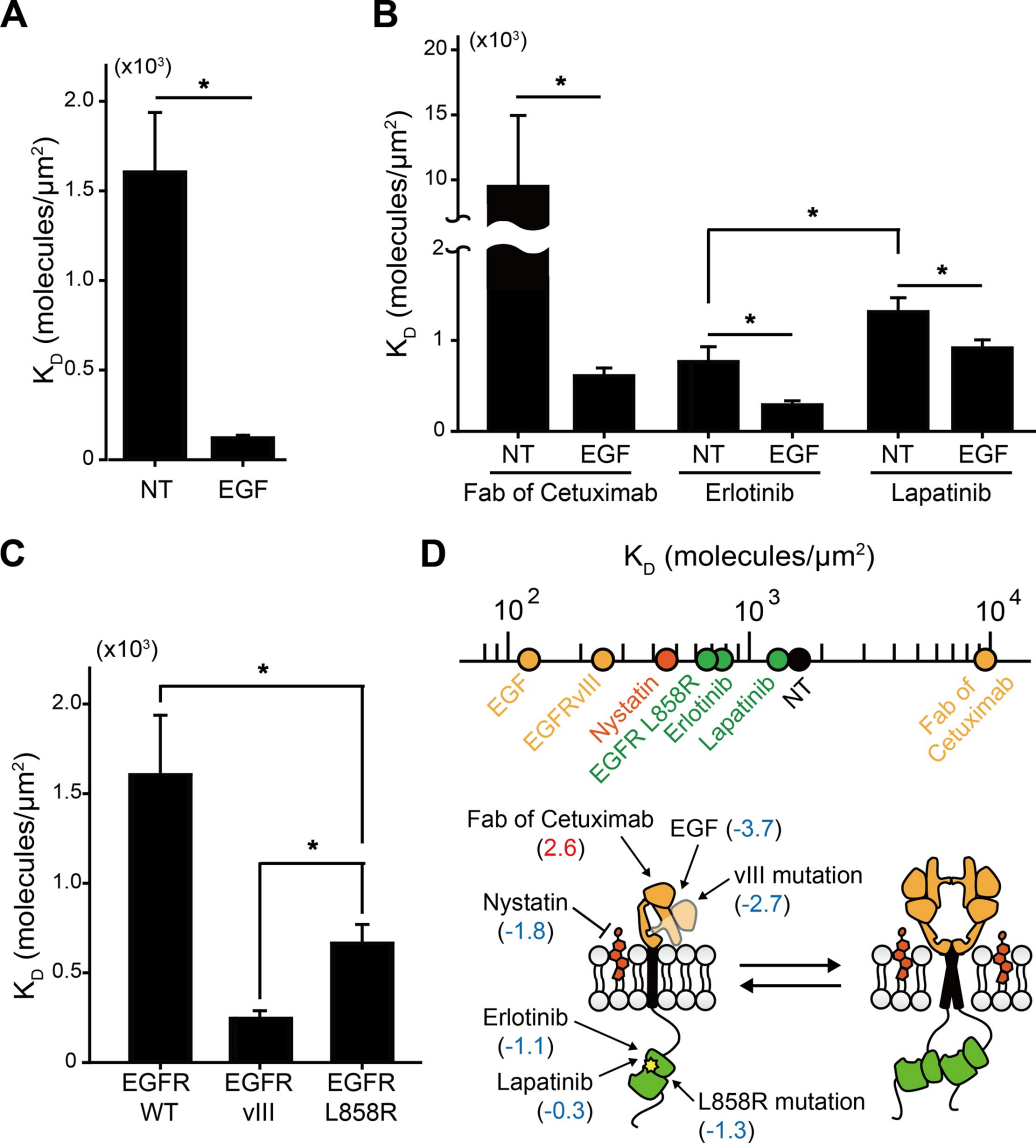
EGFR dimerization is distinctively regulated by various molecular perturbations in the membrane of living cells. (A–B) K_D_ values of EGFR homodimerization measured with and without EGF under the treatment of nonnatural ligands, including a Fab fragment of cetuximab and two types of tyrosine kinase inhibitors, erlotinib and lapatinib, in serum-starved COS7 cells. (C) K_D_ values of homodimerization for EGFR WT (the same data for NT in panel A), EGFRvIII, and EGFR L858R. The error bars represent the SEM at the single-cell level (*n* > 10). All the measurements were performed in a serum-free DMEM at 37 °C. *p < 0.05 (Student *t* test). (D) A scale mapping K_D_ values of EGFR homodimerization under various molecular perturbations. The yellow and green dots indicate the perturbations to EGFR ECD and ICD, respectively, and a black dot indicates no perturbation. Each perturbation site is displayed in the illustration, representing the reaction of EGFR pre-homodimerization with log2 fold change values compared with the K_D_ without perturbation. DMEM, Dulbecco's Modified Eagle Medium; ECD, extracellular domain; EGF, epidermal growth factor; EGFR, epidermal growth factor receptor; Fab, fragment antigen-binding; ICD, intracellular domain; NT, not treated; WT, wild type.

The ability of the in vivo K_D_ measurement using Co-II led us to explore the simple but currently unresolved question of how much the K_D_ values of EGFR dimerization decrease upon epidermal growth factor (EGF) stimulation. Above all, we confirmed that the Co-II system did not perturb ligand-induced receptor activation (S8 Fig). The K_D_ values of EGFR homodimerization determined by Co-II were 122 ± 14 and 1,606 ± 332 molecules/μm^2^ with and without EGF, respectively, in DMEM without serum at 37 °C (Fig 3A and S9 Fig). The decrease in K_D_ produced by EGF was approximately 13.2-fold. The effects of nonnatural EGFR ligands on EGFR dimerization were further examined. First, we measured the K_D_ of EGFR dimerization in the presence of the fragment antigen-binding (Fab) of cetuximab, which blocks the extended conformation of EGFR extracellular domain (ECD) [24]. As a result, the Fab fragment of cetuximab substantially impaired EGFR dimerization with and without EGF (610 ± 86 and 9,507 ± 5,450 molecules/μm^2^, respectively) (Fig 3B). The incomplete inhibition of EGF-induced EGFR dimerization by the Fab fragment might be due to the reduced affinity of the Fab fragment toward EGFR (about 2 nM), which is an order of magnitude lower than that of EGF for high-affinity binding (<0.2 nM) [24,25]. We also measured the K_D_ of EGFR dimerization after treatment with erlotinib or lapatinib, which target an ATP binding pocket in the intracellular domain (ICD) of EGFR [26] (Fig 3B and S10 Fig). Erlotinib reduced the K_D_ of EGFR dimerization without EGF, while lapatinib exerted an insignificant effect. By contrast, the inhibition potency of EGF-induced EGFR dimerization was significantly higher in lapatinib. These results indicate that erlotinib and lapatinib have different preferences on the active and inactive EGFR conformations, consistent with recent molecular structures revealed by cryo-electron microscopy (cryo-EM) [27].

Next, we explored the effects of intramolecular changes on the K_D_ of EGFR dimerization utilizing two EGFR mutants frequently found in various cancers, EGFRvIII and EGFR L858R [28]. The K_D_ values for EGFRvIII and EGFR L858R dimerization were significantly decreased by an average of approximately 6.5- and approximately 2.4-fold compared with that for EGFR WT, respectively (Fig 3C and S10 Fig). These decreased K_D_ extents indicate that these oncogenic mutants form a substantial level of dimers at their physiological expression levels in cancer, consistent with previous reports that their ligand-independent activity is derived by enhanced dimerization in cancer [29,30].

A scale mapping the K_D_ values of EGFR pre-homodimerization from the various inter- and intramolecular perturbations to EGFR was drawn (Fig 3D). Interestingly, the K_D_ values induced by perturbations to EGFR ECD tended to span a much broader range than that to EGFR ICD. Unliganded ECD conformation of EGFR has been previously controversial [11,18,30], so the contribution of EGFR ECD to EGFR pre-homodimerization was unclear. Our quantitative comparison of the K_D_ values provides direct evidence that EGFR ECD contributes more critically to EGFR pre-homodimerization than EGFR ICD does, which is consistent with a recent report displaying the dynamic conformational changes of unliganded EGFR ECD using solid-state NMR [31].

### EGFR and β2-AR homodimerizations are differentially regulated by membrane microenvironment in living cells

**Fig 4:**
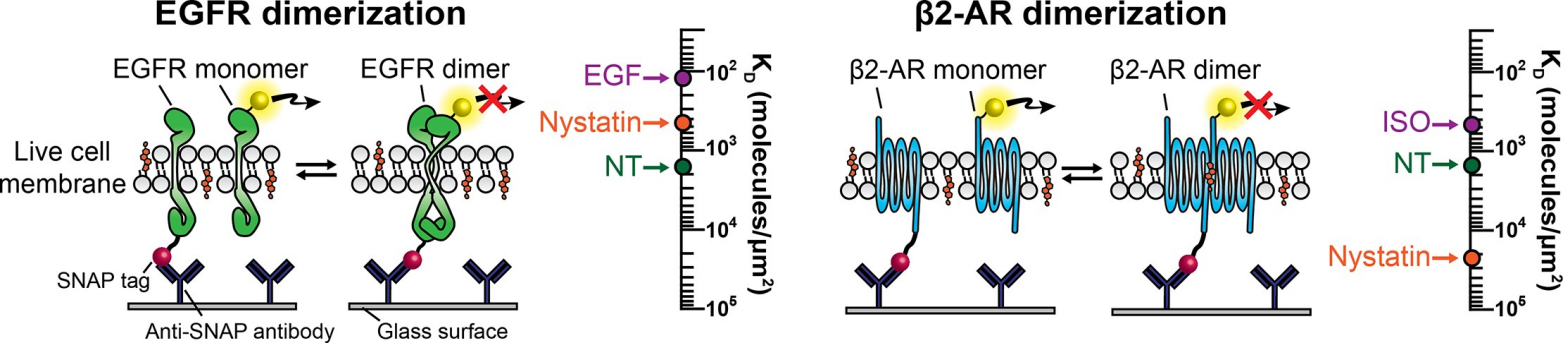
Comparison of equilibrium dissociation constants of EGFR and β2-AR homodimerization under ligand treatment and cholesterol sequestration. K_D_ values of EGFR and β2-AR homodimerizations were determined by Co-II under the existence of their ligands (EGF and ISO, respectively) and the sequestration of cholesterol in a plasma membrane. The scale mapping K_D_ values for their homodimerizations are displayed for direct comparisons. β2-AR, beta-2 adrenergic receptor; EGF, epidermal growth factor; EGFR, epidermal growth factor receptor; ISO, isoproterenol; NT, not treated; SNAP, SNAP-tag.

Recently, the β2-AR homodimer was probed using proximity-based methods, including bioluminescence resonance energy transfer (BRET), although its existence still has been controversial because of methodological concerns [32,33]. Using Co-II, we determined the K_D_ of the β2-AR pre-homodimerization in a live COS7 cell without serum (1,508 ± 145 molecules/μm^2^), which suggests the existence of a mixture of both β2-AR monomer and homodimer at a typical physiological expression level in living cells [34], although the K_D_ value measured in situ using Co-II is about 3-fold lower than the value measured in vitro using proteoliposome, likely due to the effect of the microenvironmental context of a plasma membrane [35,36]. Interestingly, the K_D_ of the β2-AR pre-homodimerization is similar to that of EGFR pre-homodimerization (1,606 ± 332 molecules/μm^2^), which led us to further investigate the differences between the homodimers of these two receptors in distinct receptor classes. The addition of isoproterenol decreased the K_D_ of the β2-AR dimerization by about 3.3-fold (462 ± 82 molecules/μm^2^), unlike EGF, which decreased the K_D_ of the EGFR dimerization by about 13.2-fold (122 ± 14 molecules/μm^2^). This result is possibly derived from the lack of an explicit structural interface for the β2-AR dimerization such as the dimerization arm of EGFR extended by EGF [11,37]. Both EGFR and β2-AR have been previously reported to be regulated by membrane microenvironment, such as a cholesterol [38,39]. We compared the K_D_ values of the homodimerizations of the two receptors after sequestrating cholesterol in a plasma membrane. Surprisingly, the dimerization of β2-AR was markedly disrupted by nystatin (22,378 ± 4,283 molecules/μm^2^), whereas that of EGFR was significantly enhanced (453 ± 95 molecules/μm^2^), indicating that EGFR and β2-AR homodimerizations are differentially regulated by the membrane microenvironment. A scale mapping the K_D_ values of the homodimerizations of these two receptors under ligand treatment and cholesterol depletion was drawn in Fig 4.

## Discussion

Co-II analyzes membrane protein interactions based on their inherent diffusivity instead of their proximity, which is utilized for prevalent methods. This principle of Co-II liberates concentration dependency, which is critical when proximity is used as an indicator for the physical interaction, because random collision between noninteracting proteins can frequently occur at high concentration. Co-II provides reliable data even at the high density in a crowded membrane of living cells, as no interaction between EGFR and PMT was observed even at a saturated expression level (K_D_ = 26,890 ± 2,724 molecules/μm^2^) (S11 Fig). Furthermore, Co-II is conceptually independent of the tag orientation on the proteins because the intrinsic property of a protein itself is the subject of the measurement in Co-II, whereas the tag is the subject in the proximity-based methods [8] (S10 Fig). Therefore, the bona fide analysis using Co-II could provide unprecedented quantitative information regarding membrane protein interactions affected by natural ligands, drugs, mutations, and microenvironmental changes in a single living cell (Figs 3 and 4).

Although single-molecule trajectories contain convoluted information regarding multiple molecular processes, the interpretation of protein diffusion has been subjective; the changes of diffusion coefficient were interpreted as one distinct molecular process based on a theoretical assumption, without thorough experimental verification [23,40]. Furthermore, transient colocalization among single-molecule trajectories has been presumed as their molecular interaction, even though the colocalization is only a necessary condition for the physical interaction because the localization accuracy of fluorescent proteins is not sufficient to resolve direct molecular interactions [41,42]. Although two-color quantum dot tracking has circumvented this problem by observing colocalizing trajectories with a correlated motion, the probability of physical interaction between two sparsely visualized fluorescently labeled proteins is extremely low, which restrains this approach from directly assessing the number of interacting molecules to determine equilibrium constants [43]. Thus, the lacking objectiveness for biologically interpreting trajectory data has limited the application of SPT to specific research and requires elaborate experimental controls to prevent the misinterpretation of data. These problems are mainly derived from the fact that the natural change in the diffusion coefficient made by the interaction between two diffusing proteins is marginal. However, the objective deconvolution of interaction information from single-molecule trajectories becomes possible with Co-II, because the change in diffusion coefficient by the interaction is represented by an order of magnitude difference in the diffusion coefficient (from about 0.2 μm^2^/s to about 0.008 μm^2^/s) and the change appears by a controllable trigger, the antibody addition. The application of Co-II gets retarded as the diffusion of a prey is slowed, due to the classification error between mobile and immobile trajectories. The diffusion coefficient of a prey up to 0.04 μm^2^/s can be applied for Co-II using mEos3.2, considering the full width at half maximum of the log distributions of EGFR and β2-AR diffusion coefficients obtained by using mEos3.2.

Compared with previous methods to detect protein–protein interactions by using protein immobilization and the bulk measurement of fluorescent intensity [44], there are several major advantages in Co-II. First, it enables the direct visualization of single-protein interactions in living cells, which makes it possible to perform single-molecule research in living cells. Because membrane proteins in the plasma membrane of living cells coexist at very high concentration and constantly flow, the spatiotemporal positions of individual proteins cannot be accurately determined, even using super-resolution microscopy. Co-II overcame this concentration problem in the membrane of living cells by utilizing the dimension of a protein’s intrinsic diffusivity in addition to space and time dimensions. Second, high measurement sensitivity is achieved from the large number of single-molecule data. It was possible to precisely probe about 3% of the co-immobilized EGFR fraction using more than 10,000 single-molecule trajectories. This high sensitivity cannot be reached by the bulk fluorescence intensity, which fluctuates at high level and is vulnerable to photobleaching. This sensitivity issue becomes critical as the protein–protein interaction of interest is weaker or more transient. Third, quantitative information is derived from the counting of single molecules, enabling robust and precise quantification with a linear dynamic range. Furthermore, Co-II does not suffer from photobleaching because diffusivity, not fluorescent intensity, is the measurement, which generates reliable data even with multiple measurements. Lastly, it might provide relative stoichiometry information. We analyzed the frequency of stopping EGFR in the vicinity of the immobilized one, which enables us to infer the distribution of the oligomer size (S12 Fig). This stoichiometry analysis implies that EGFR dimer is a major population, with a small portion of oligomers induced by EGF. More extensive experiments might be required to verify whether the recently reported EGFR tetramers exist at a significant level [45,46].

K_D_ values over a wide range, encompassing both strong and weak interactions, can be analyzed using Co-II simply by controlling the expression of bait proteins. Eq 3 in the Materials and methods section provides the optimal expression range of a bait protein for determining K_D_ (S13 Fig). The interactions more than 1,000 times stronger or 10 times weaker than EGFR pre-homodimerization can be resolved. The measurement of high K_D_ values for weak interactions becomes possible due to the ability to capture the rapid transient interactions between membrane proteins using SPT [47].

Because Co-II utilizes the immobilization of the one of the reactants, the measured reaction rate for homodimerization should be equal to the true rate divided by two, according to the Smoluchowski reaction rate, if the reaction of interest is diffusion controlled [48]. Although biochemical reaction kinetics on the plasma membrane might be affected by the crowdedness or the microdomains of the plasma membrane, which contribute to proteins’ diffusivity, it is not clear whether receptor dimerization is actually diffusion controlled. In case of EGFR, the conformational change of EGFR ECD from a tethered form to an extended form is crucial for its dimerization [49], implying that the activation energy is a major factor for EGFR dimerization. Furthermore, EGF binding marginally affects the diffusion coefficient of EGFR, according to the Saffman-Delbruck model [50] and our measurement. Care must be taken to interpret K_D_ values measured by Co-II, considering whether the reaction of interest is governed by activation energy or diffusion. Conversely, this bias might be useful to characterize whether the reaction process is activation controlled or diffusion controlled.

Co-II should not be limited to statistically analyzing K_D_ at the ensemble level. The power of Co-II can be expanded to provide dynamic interaction constants such as a dissociation constant (k_off_) and an association constant (k_on_) at the single-molecule level and reveal the single-molecule heterogeneity of membrane protein interactions in living cells. By obtaining long trajectories using a photoswitchable organic dye, Alexa Fluor 647, we directly visualized the dissociation process (the mobile-immobile-mobile transition) of single-molecule EGFR pre-homodimerization (S4 Fig). Although the probability of observing the process was substantially low (0.001) due to the insufficient duration of trajectories obtained by Alexa Fluor 647, the measured k_off_ value (about 1.2 s^−1^) was similar to the previous report measured by the two-color colocalization of quantum dot trajectories [43]. Repetitive interactions of a single mobile prey with immobilized baits can be observed if a fluorescent probe or a nanoparticle that yields sufficiently long trajectories is utilized.

Recently, the distinct regulation of ErbB3 phosphorylation by the interaction with EGFR upon the stimuli of different ligands was reported [51], in which HER3 dimerization and clustering with EGFR are differentially controlled by different ligands. Along with this finding, our result that EGFR and β2-AR homodimerizations are differentially regulated by cholesterol demonstrates that the microevironment of the plasma membrane is critically involved in their activation mechanism in living cells, which lies on shared context with previous reports [52,53,54]. These observations together strongly suggest that receptor activation is differentially regulated by both the intramolecular conformation and the microenvironment of the plasma membrane in living cells. Co-II should be useful to elucidating the dynamic changes of membrane protein interactions in the diverse physiological contexts of living cells and understanding the precise regulation of receptor activation in the membranes of living cells.

## Materials and methods

### Plasmids, antibodies, and reagents

To construct the mEos3.2 fusion protein at the N terminus of EGFR, we subcloned human EGFR into the pcDNA3.1 vector (V800-20, Invitrogen) with the following primers 1–4. Then, mEos3.2 extracted from pEGFP-N1/mEos3.2, a kind gift from Dr. Tao Xu (Chinese Academy of Science), was inserted between the signal and mature peptide of EGFR with the following primers 5–6. To construct SNAP-tagged EGFR, the SNAP tag gene from the pSNAPf vector (N9183S, New England Biolabs) was subcloned into pcDNA3.1/mEos3.2-EGFR with the following primers 7–8. The SNAP-tagged EGFRvIII (SNAP-EGFRvIII) and EGFR L858R constructs (SNAP-EGFR L858R) were obtained by replacing the EGFR WT gene from pcDNA3.1/SNAP tag-EGFR with the EGFRvIII and EGFR L858R genes using the following primers 9–10 and 11–12, respectively. To construct the mEos3.2-tagged InsR at the C terminus, we first subcloned the InsR gene, a kind gift from Ingo Leibiger (Karolinska Institutet, Sweden), into pcDNA3.1/mEos3.2-His at the N terminus of mEos3.2 with the following primers 13–14. To construct SNAP-tagged β2-AR, we subcloned the SNAP tag gene into the N terminus of β2-AR with the signal peptide from hemagglutinin to enhance membrane localization. The corresponding templates were obtained from Matthew Meyerson (Addgene plasmid #11011 for EGFR WT; Addgene plasmid #11012 for EGFR L858R), Alonzo Ross (Addgene plasmid #20737 for EGFRvIII), and Robert Lefkowitz (Addgene plasmid #14697 for β2-AR). All the other plasmids, including PMT-mEos3.2, EGFR-mEos3.2, EGFRvIII-mEos3.2, EGFR L858R-mEos3.2, ErbB2-mEos3.2, ErbB3-mEos3.2, and β2-AR-mEos3.2, were prepared as previously described [47].

Primer 1: 5′-CGCAAATGGGCGGTAGGCGTG

Primer 2: 5′-CCGCGGTTGGCGCGCCAGCCCGACTCGCCGGGCAGAG

Primer 3: 5′-GGCGCGCCAACCGCGGCTGGAGGAAAAGAAAGTTTGC

Primer 4: 5′-AGCTTTGTTTAAACTTATGCTCCAATAAATTCACTGCT

Primer 5: 5′-GGCGCGCCACATCATCACCATCACCATATGAGTGCGATTAAGCCAGAC

Primer 6: 5′-TCCCCGCGGCCCTCCACTCCCACTTCGTCTGGCATTGTCAGGCAA

Primer 7: 5′-GGCGCGCCACATCATCACCATCACCATATGGACAAAGACTGCGAAATG

Primer 8: 5′-TCCCCGCGGCCCTCCACTCCCACT ACCCAGCCCAGGCTTGCCCAG

Primer 9: 5′-TCCCCGCGGCTGGAGGAAAAGAAAGGTAAT

Primer 10: 5′-AGCTTTGTTTAAACTCATGCTCCAATAAATTCACT

Primer 11: 5′-TCCCCGCGGCTGGAGGAAAAGAAAGTTTGC

Primer 12: 5′-AGCTTTGTTTAAACTCATGCTCCAATAAATTCACT

Primer 13: 5′-CGGGATCCATGGCCACCGGGGGCCGGCGG

Primer 14: 5′-GCTCTAGAACTCCCGGAAGGATTGGACCGAGGCAA

The antibodies and reagents were obtained from the following vendors: the mAb 199.12 (AHR5072) and Alexa Fluor 647–conjugated anti-mouse antibody (A21235) were obtained from Invitrogen; both mAb 528 (sc-120) and mAb R-1 (sc-101) were obtained from Santa Cruz; the SNAP tag antibody (CAB4255), rabbit anti-mouse IgG (31194), biotin-conjugated EGFR antibody (MA5-12872), and anti-6x His tag antibody (MA1-21315) were obtained from Thermo Scientific; the anti-mEos3.2 antibody (A010-mEOS) was purchased from Badrilla; the anti-phosphorylated EGFR antibody (Y1068, ab32430) was obtained from Abcam; the anti-actin antibody (691001) was obtained from MP Biomedicals; cetuximab was obtained from Merck Serono; erlotinib and lapatinib were obtained from Selleckchem; and EGF (E9644), nystatin (N6261), and isoproterenol (I5627) were purchased from Sigma-Aldrich.

The cetuximab Fab fragment was generated from an intact antibody using a Fab preparation kit (44685, Pierce), and cetuximab was labeled with Alexa 647 dye using the Alexa Fluor 647 Antibody Labeling Kit (A20186, Thermo Scientific).

CF660R, succinimidyl ester (92134, Biotium), was reacted with BG-NH2 (S9148S, New England Biolabs) in dimethylformamide while shaking at 30 °C overnight according to the manufacturer’s instructions. The solvent was vacuum-evaporated and the product was dissolved in distilled water after purification by HPLC.

### Cell culture and transfection

COS7, HEK293, and HeLa cells were obtained from American Type Culture Collection (ATCC) and cultured in DMEM (Lonza) supplemented with 10% FBS (Gibco) at 37 °C, 5% CO_2_, and 95% humidity. CHO-K1 cells (ATCC) were cultured in DMEM/F-12 1:1 modified medium (Thermo Scientific) supplemented with 10% FBS at 37 °C, 5% CO_2_, and 95% humidity. The cells were transfected using lipofectamine LTX (Invitrogen) according to the manufacturer’s instructions.

### Sample preparation

Glass coverslips were washed in chloroform/methanol (50/50) for 24 h and stored in ethanol. After drying, the coverslips were oxidized in a plasma chamber (Femto Science) for 5 min and then incubated in a closed jar containing a silanization solution (methanol, 4.5% deionized water, 0.9% acetic acid, 2.5% 3 mercapto-pro-pyulrimethoxy silane [S10475, Fluorochem]) overnight at 4 °C. After washing three times with PBS, the coverslips were reacted with 0.1 mg/mL of maleimide-activated neutravidin protein (31007, Thermo Scientific) with 50 μg/mL fibronectin (F2006, Sigma-Aldrich) for 1 h at room temperature. Subsequently, a biotin-conjugated anti-rabbit IgG H + L antibody (ab7089, Abcam) or a biotin-conjugated anti-mouse IgG Fc antibody (A16088, Invitrogen) was added, and the coverslips were incubated for 1 h at room temperature. The cells were seeded on the prepared coverslip coated with the secondary antibody that captures the anti-bait antibody. The cells on the coverslips were maintained in phenol red–free DMEM (Thermo Scientific) during imaging. The cells were starved for 4 h and then treated with either 10 μg/mL cetuximab Fab fragment for 1 h, 5 μM tyrosine kinase inhibitors for 4 h, 10 μg/mL nystatin for 1 h, 10 nM EGF for 1 h, or 1 μM isoproterenol for 1 h. The immobilized fractions of a prey protein were marginally changed during the bait immobilization under these conditions. The 100 μg/mL anti-bait antibody was treated immediately after the image acquisition without bait immobilization was finished. After 5 to 15 min of the anti-bait antibody treatment, which is sufficient for the antibody to fully penetrate the space between a cell bottom and a glass surface, the image acquisition with bait immobilization was performed in the same cells.

Alternatively, the cells can be seeded on the glass coated with the anti-bait antibody. In this case, there is no need to treat an anti-bait antibody during the imaging, which makes the experiments much simpler and facilitates cell attachment within 15 to 30 min. However, it is difficult to specifically determine the antibody-induced co-immobilized prey population at the single-cell level because a pre-immobilized prey population often exists at basal states (before treating the antibody). This problem can be resolved by analyzing data obtained from multiple cells in the absence and presence of the antibody, providing the result at the cell-population level.

Approximately 1,000 prey trajectories were sufficient to precisely determine the co-immobilized fraction. Thus, the overexpression issue should not be the major concern, considering the typical endogenous levels of EGFR in relevant cell lines (typically, between 10^4^ and 10^6^ molecules), although the bait protein is expressed at much higher levels than the prey protein.

### Microscopy and image acquisition

Co-II was performed using a homemade TIRF microscope built on an inverted microscope (IX-81, Olympus) equipped with an XY-axis automated stage (MS-2000, Applied Scientific Instrumentation) and a live-cell chamber to maintain cells on coverslips at 37 °C with 5% CO_2_ during image acquisition (Chamlide TC-A, Live Cell Instrument). A 405-nm laser (DL-405-120, Crystal Laser), a 561-nm laser (for a red form of mEos3.2 excitation, YLK 6150T, Lasos) and a 642-nm laser (for Alexa 647 excitation, VFL-P-1000-642, MPB Communications) were aligned for TIRF illumination with an oil-immersion objective lens (APON 100XOTIRF/1.49, Olympus). Emission light separated by a dichroic mirror (ZET405/488/561/647m, Chroma) and emission filters (T635lpxr and ET655lp, Chroma) equipped in TuCam (Andor Technology) was collected using an electron multiplying charge-coupled device (EM-CCD) camera (iXon Ultra 897, Andor Technology). To obtain the diffusion-coefficient distributions of membrane proteins, membrane proteins conjugated with mEos3.2 were photoactivated with a 405-nm laser under TIRF illumination for 100 to 1,000 ms, depending on their expression level, with an intensity of 0.2 to 0.5 W/cm^2^ measured at the back focal plane of the objective. Then, 200 frames were imaged using a 561-nm laser with an intensity of 20 to 30 W/cm^2^ at a frame rate of 20 Hz. This activation-imaging cycle was repeated 4 to 10 times to acquire a sufficient number of trajectories. The image for CF660R was acquired using a 647-nm laser with an intensity of 30 to 40 W/cm^2^ at a frame rate of 20 Hz. All instrument operations and data acquisition were controlled by MetaMorph (Molecular Devices) and custom plug-ins written in MATLAB (MathWorks). The algorithm of multiple particle tracking was previously described [44].

### Determination of immobilized fractions from single-molecule trajectories

Two-dimensional diffusion coefficients were calculated from the MSD,

$$MSD=4Dt+4e^{2}$$

where MSD(Δt) = E((x_*t*+Δt_−x_*t*_)^2^+(y_*t*+Δt_−y_*t*_)^2^) and (x_*t*_,y_*t*_) are the Cartesian coordinates of particles at the t^th^ point of their trajectory, D is the diffusion coefficient, and *e* is the localization error. Trajectories with a duration longer than eight frames were used to calculate the diffusion coefficients using four time lags of MSD.

The immobilization criteria were objectively determined based on the localization error distribution of fluorophores, *σ*^2^/Δ*t*, where σ is the 95% upper bound of localization error (30 to 60 nm) and Δt is the time gap between frames (about 53 ms). Immobilized fractions were calculated by counting single-molecule trajectories classified into mobile and immobile subpopulations. The utilization of an anomalous diffusion equation clearly separated mobile and immobile subpopulations in the distributions, but the determined immobilized fractions based on free and anomalous diffusion were equivalent.

The duration of trajectories is important for the accurate classification of single-molecule trajectories. Depending on the dissociation rate of the complex, the duration of co-immobilization could be shorter than that of the trajectories, which yields the average diffusion coefficient of mobile and immobile states that appear in one trajectory. This effect may result in underestimation of the co-immobilized fraction. However, because short trajectories tend to produce broad diffusion-coefficient distributions, the classification between the mobile and immobile states becomes difficult as trajectories are short. Thus, the control of the trajectory duration by increasing the frame rate with a brighter dye is beneficial for investigating very weak interactions.

### Calculation of equilibrium dissociation constants using Co-II

An equilibrium dissociation constant, K_D_, in Co-II is defined by

$$K_{D}=\frac{\left[R][I\right]}{\left[RI\right]}$$ (1)

where [R], [I], and [RI] are the absolute concentrations of a dissociated mobile prey protein, *R*, a dissociated immobilized bait protein, *I*, and the associated immobile *RI* at equilibrium, respectively.

Because the co-immobilized fraction of a prey protein at equilibrium, ρ_R_, provides the ratio of a complex, the equation for K_D_ becomes

$$K_{D}=\frac{\left({\left[R\right]}_{0}-{\left[R\right]}_{0}\rho_{R})({\left[I\right]}_{0}-{\left[R\right]}_{0}\rho_{R}\right)}{{\left[R\right]}_{0}\rho_{R}}$$ (2)

where [R]_0_ and [I]_0_ are the total concentrations of the R and I proteins, respectively.

When the amount of the immobilized bait protein is excessive compared with that of the complex, the absolute concentration of the bait protein after reaching dynamic equilibrium can be assumed to be equal to the initial concentration, [I]_0_. Thus, the second-order bimolecular reaction becomes a pseudo-first-order reaction, which converts [R]/[RI] into a dimensionless unbound/bound ratio of the prey to the bait proteins at equilibrium, ρ_R_, after immobilizing the bait protein in Co-II. Thus, the equilibrium dissociation constant equation becomes

$$K_{D}=\frac{\left(1-\rho_{R}\right){\left[I\right]}_{0}}{\rho_{R}}$$ (3)

ρ_R_ is determined from the diffusion-coefficient distribution of the prey protein before and after the immobilization of the bait. Considering the immobile subpopulation of the prey existing before the antibody-induced immobilization of the bait, which is irrelevant to co-immobilization, the co-immobilized fraction is calculated by the decreased fraction of the mobile prey subpopulation after the immobilization of the bait.

$$\rho_{R}=1-P_{\mathrm{mobile},\;\mathrm{after}}/P_{\mathrm{mobile},\;\mathrm{before}}$$ (4)

where P_mobile, before_ and P_mobile, after_ are the mobile fractions of the prey before and after the immobilization of the bait, respectively.

The concentration of the antibody-induced immobilized bait, [I]_0_, is calculated by

$${\left[I\right]}_{0}={\left[B\right]}_{0}\times B_{\mathrm{mobile},\;\mathrm{before}}{\times \varepsilon }_{I}$$ (5)

where [B]_0_ is the total concentration of the bait, B_mobile, before_ is the mobile fraction of the bait before immobilization, and ε_I_ is the antibody-induced bait immobilization efficiency.

For homodimerization, the mobile fractions of the prey and bait before the immobilization are the same, which makes [I]_0_ = [B]_0_×P_mobile, before_×ε_I_, and

$$K_{D}=\frac{P_{\mathrm{mobile},\;\mathrm{before}}P_{\mathrm{mobile},\;\mathrm{after}}}{P_{\mathrm{mobile},\;\mathrm{before}}-P_{\mathrm{mobile},\;\mathrm{after}}}{\left[B\right]}_{0}\varepsilon_{I}$$ (6)

Because antibody-induced bait immobilization efficiency is independent of the bait concentration, the average of the above equation becomes

$$E\left(K_{D}\right)=E\left(\frac{P_{\mathrm{mobile},\;\mathrm{before}}P_{\mathrm{mobile},\;\mathrm{after}}}{P_{\mathrm{mobile},\;\mathrm{before}}-P_{\mathrm{mobile},\;\mathrm{after}}}{\left[B\right]}_{0}\right)E\left(\varepsilon_{I}\right)$$ (7)

E(ε_I_) is about 0.93 for SNAP-EGFR and SNAP-β2-AR using an anti-SNAP antibody.

Therefore, only the diffusion-coefficient distribution of a prey and the concentration of a bait are required to determine K_D_ for homodimerization at the single-cell level using Co-II.

There is a possibility that the bait–bait interaction might titer out the available monomeric bait molecules, which makes the [B]_0_ overestimated. However, bait–bait interactions are typically far weaker than the bait–antibody interaction. Thus, once the dissociated bait monomer from the transient bait dimer is captured by the antibody coated on a glass surface, the antibody-captured bait monomer should no longer diffuse and associate with the other bait monomers, because almost every bait monomer is immobilized by the antibody excessively coated on a glass surface. If the interaction of interest is strong, more waiting time after the antibody addition might be required to fully dissociate the bait proteins.

### Absolute quantification of the expression level of membrane proteins

Cells were illuminated by a 647-nm laser to detect SNAP-tagged proteins labeled with CF660R at the maximum power not resulting in photobleaching in 10 consecutive TIRF image acquisitions. Total expression of SNAP-tagged proteins was quantified from the intensity of fluorescence inside a cell subtracted by that outside a cell, using ImageJ. After photobleaching up to the density at which individual fluorophores could be resolved, only fluorescent molecules displaying a single photobleaching step were sorted in the images obtained at the same power used for measuring total expression level. The intensity of the sorted single fluorophore was measured by the area under a Gaussian-fit curve. The median intensity of single fluorophores was used to avoid outliers. The absolute concentration of the fluorophore was calculated by dividing the total fluorescence intensity by the single molecule intensity. The concentration of SNAP-EGFR on the COS7 cell surface was determined by normalizing the total fluorescence intensity by the single-molecule intensity. The total number of SNAP-EGFRs was corrected, considering the proportion of nonfluorescent CF660R that is immobilized but not detected. The fluorescent portion of CF660R was determined by labeling SNAP-tagged EGFR with an Alexa647-conjugated anti-EGFR antibody whose fluorescent dye-to-antibody ratio was predetermined in vitro.

### Determination of immobilized fractions according to the expression of EGFR

Cells expressing mEos3.2-EGFR were sorted 48 h after transfection into three populations of low, middle, and high expression using a green form of mEos3.2 (MoFlo XDP, Beckman Coulter). To measure immobilized fractions, each population was seeded onto pre-cleaned glasses, and the ratio of the immobilized fractions in each population before and after anti-mEos3.2 antibody treatment was determined.

### In situ immunostaining of EGFR phosphorylation

Cells expressing SNAP-EGFR were seeded onto pre-cleaned coated glass and treated with an anti-SNAP antibody to immobilize SNAP-EGFR. To stimulate EGFR, 10 nM EGF was added for 5 min. Cells were lysed with RIPA buffer and washed three times with PBS. The remnants of immobilized SNAP-EGFR were confirmed by fluorescence imaging. The coverslips were treated with anti-phospho EGFR and Alexa 647–labeled secondary antibodies for 30 min. After washing, fluorescence images were obtained to assess the EGFR phosphorylation levels in media supplemented with 5 mM protocatechuic acid (PCA, sc-205818, Santa Cruz), 0.5 U/mL protocatechuate-3,4-dioxygenase (PCD, P8279, Sigma-Aldrich), and 1 mM β-mercaptoethylamine (MEA, 30070, Sigma-Aldrich).

## Supporting information

S1 Data Data underlying Figs 1–3 and S1, S2, S3, S7, S9 and S10 Figs. (XLSX)

S1 Fig Complete immobilization of bait membrane protein in Co-II assay. (A) Illustration and immobilization efficiency of the three types of immobilization systems for EGFR-mEos3.2 of varying heights from the glass surface in COS7 cells. Each dot represents single-cell data, and the red solid lines indicate the average of the immobilized fractions obtained from multiple cells (*n* > 10). (B) COS7 cells expressing mEos3.2-EGFR were sorted into three parts based on the expression level (low, middle, and high) using the mEos3.2 fluorescence intensity. (C) The immobilized fractions of mEos3.2-EGFR before (empty circle) and after mEos3.2 antibody treatment (filled circle) were constant across all three levels of EGFR expression (low: blue circles, middle: red circles, high: green circles), as calculated by the mEos3.2 fluorescence intensity from TIRF images. Each dot represents single-cell data. (D–F) The immobilized fraction of EGFR before and after treatment with three different anti-EGFR antibody clones with different EGFR binding epitopes: mAb 199.12 (D), mAb R-1 (E), and mAb 528 (F). (G) The immobilized fraction of EGFR before and after anti-SNAP antibody treatment in cells expressing SNAP-EGFR. (H–I) The immobilized fraction of EGFR before and after treatment with an anti-mEos3.2 antibody in cells expressing mEos3.2-EGFR (H) and EGFR-mEos3.2 (I). Each dot represents single-cell data, and the red solid lines indicate the average of the immobilized fractions obtained from multiple cells (*n* > 10). *p < 0.05 (Student *t* test). EGFR, epidermal growth factor receptor; mEos3.2, monomeric Eos fluorescent protein variant 3.2; n.s., nonsignificant difference; SNAP, SNAP-tag; TIRF, total internal reflection fluorescence.
(TIF)

S2 Fig The effect of antibody-induced immobilization on bait proteins. (A) Alexa Fluor 488–labeled anti-SNAP antibody was treated to a COS7 cell expressing SNAP-EGFR (non-labeled) seeded on a cleaned glass to visualize the process of the antibody penetration between the cell bottom and the glass surface. The antibody was fully penetrated across the entire cell surface within 10 min. (B) The anti-SNAP antibody was treated to a COS7 cell expressing SNAP-EGFR labeled by BG-CF660R seeded on the anti-rabbit secondary antibody-coated glass to observe the effect of the antibody-induced SNAP-EGFR immobilization on the distribution of EGFR on the plasma membrane. No significant change in EGFR distribution on the plasma membrane was detected. (C) FRET experiments were performed to examine whether the cross-linking of SNAP-EGFR is produced by the surface immobilization using anti-SNAP antibody. BG-Cy3 and BG-Cy5 were treated at 1:1 ratio on COS7 cells expressing SNAP-EGFR seeded on the anti-rabbit secondary antibody-coated glass. Both Cy3 (donor) and Cy5 (acceptor) channels were monitored with a donor-only excitation. Then, the cells were treated with EGF or anti-SNAP antibody. FRET ratios (acceptor/donor) were normalized to analyze the relative changes in FRET ratios by the treatments (*n* > 5). No significant cross-linking was observed by the anti-SNAP antibody induced SNAP-EGFR immobilization. Scale bars, 5 μm. BG, benzyl guanine; EGF, epidermal growth factor; EGFR, epidermal growth factor receptor; FRET, fluorescence resonance energy transfer; SNAP, SNAP-tag.
(TIF)

S3 Fig Molecule-specific immobilization in the plasma membrane of a living cell. (A) Diffusion-coefficient distributions of SNAP-EGFR and β2-AR-mEos3.2 before (black lines) and after anti-EGFR antibody treatment (red lines). (B) Diffusion-coefficient distributions of EGFR-mEos3.2 and SNAP-β2-AR before (black lines) and after anti-SNAP antibody treatment (red lines). β2-AR, beta-2 adrenergic receptor; EGFR, epidermal growth factor receptor; mEos3.2, monomeric Eos fluorescent protein variant 3.2; SNAP, SNAP-tag.
(TIF)

S4 Fig Molecular colocalization of co-immobilized SNAP-EGFR with immobilized mEos3.2-EGFR. The red line indicates a single molecule trajectory of SNAP-EGFR labeled with Alexa Fluor 647 (the prey), and the white dots represent antibody-induced immobilized mEos3.2-EGFR (the bait). To acquire long trajectories to observe the transition of mobile-immobile-mobile states, we utilized benzyl-guanine–conjugated Alexa Fluor 647 instead of mEos3.2. Therefore, we immobilized mEos3.2 using anti-mEos3.2 antibody instead of the SNAP tag. The temporarily immobilized SNAP-EGFR was colocalized with the antibody-induced immobilized mEos3.2-EGFR within 30 nm. Scale bar, 500 nm. EGFR, epidermal growth factor receptor; mEos3.2, monomeric Eos fluorescent protein variant 3.2; SNAP, SNAP-tag.
(TIF)

S5 Fig Correction for the measurement of the expression level of SNAP-EGFR. The fluorescent SNAP-CF660R-EGFR ratio was determined. TIRF image of the total expression and single-molecule fluorescence of SNAP-CF660R-EGFR and cetuximab-Alexa Fluor 647–labeled EGFR in HeLa cells, which marginally express endogenous EGFR. Scale bar, 5 μm. The ratio between protein concentrations quantified using CF660R-SNAP and cetuximab-Alexa Fluor 647 was 0.91 ± 0.13. EGFR, epidermal growth factor receptor; SNAP, SNAP-tag; TIRF, total internal reflection fluorescence.
(TIF)

S6 Fig Cell viability before and after the Co-II assay. DIC images were taken before and after performing the Co-II assay in the same cell. Photodamage to cell morphology was undetectable. Scale bar, 5 μm. DIC, differential interference contrast.
(TIF)

S7 Fig Spatial K_D_ distribution of EGFR pre-dimerization with the different sizes of average window. (A, C, E, G) Spatial K_D_ maps of EGFR pre-homodimerization in a single living cell with different sizes of average window (1.2 μm, 1.8 μm, 2.4 μm, and 3.6 μm, repectively). Scale bar, 5 μm. (B, D, F, H) The K_D_ profiles obtained from cross sections corresponding to the red dashed lines in respective panels. (I) Standard deviation of K_D_ maps with different sizes of average window. EGFR, epidermal growth factor receptor.
(TIF)

S8 Fig Effect of immobilization on EGFR phosphorylation. SNAP-EGFR expressed in COS7 cells was immobilized onto a glass surface using an anti-SNAP antibody. The cells were lysed after mock (A) or EGF (B) treatment, resulting in SNAP-EGFR being held by the antibody coated on a glass surface. As a positive control, the cells were treated by EGF first, then immobilized onto a glass surface (C). The phosphorylation level of the remaining SNAP-EGFRs was measured using an anti-EGFR Y1068 antibody and an Alexa Fluor 488–conjugated secondary antibody. Cells with similar levels of SNAP-EGFR expression were examined to compare the phosphorylation of the remaining SNAP-EGFRs with or without EGF treatment. Scale bar, 5 μm. An enormously elevated level of phosphorylation induced by EGF was detected compared with the basal level of phosphorylation without EGF treatment, whereas a similar level of phosphorylation was observed regardless of the order of the EGF treatment and the SNAP-EGFR immobilization. EGF, epidermal growth factor; EGFR, epidermal growth factor receptor; SNAP, SNAP-tag.
(TIF)

S9 Fig Diffusion-coefficient distributions for EGFR homodimerization before and after the bait immobilization under a EGF-treated condition. (A) Diffusion-coefficient distribution of SNAP-EGFR and EGFR-mEos3.2 before (black line) and after anti-SNAP antibody treatment (red line) in a EGF-treated COS7 cell. (B) The immobilized fractions of SNAP-EGFR and EGFR-mEos3.2 before and after anti-SNAP antibody treatment. EGF, epidermal growth factor; EGFR, epidermal growth factor receptor; mEos3.2, monomeric Eos fluorescent protein variant 3.2; SNAP, SNAP-tag.
(TIF)

S10 Fig Analysis of the binding curves of Co-II assay. Co-immobilized fractions, ρ, determined from Co-II assay were analyzed from the binding curves to estimate K_D_ values (*n* ≥ 5). Because K_D_ = (1−ρ)/ρ*[I]_0_ in Co-II, we utilized a linear fit to reduce the complexity of the curve fitting using the equation, ρ/(1−ρ) = a*[I]_0_, where *a* is the slope of the linear fit (a solid red line) representing 1/K_D_, and [I]_0_ is the density of antibody-induced immobilized bait proteins. The 95% confidence intervals are shown (dashed red lines). (A–J) The analysis of the homodimerization of EGFR WT pretreated with mock (A, B), erlotinib (C, D), lapatinib (E, F), nystatin (G, H), and cetuximab (I, J) followed by mock treatment (A, C, E, G, I) or EGF treatment (B, D, F, H, J). (K, L) The analysis of the homodimerization of EGFR L858R (K) or EGFRvIII (L). (M–P) The analysis of the homodimerization of β2-AR WT pretreated with mock (M, N) or nystatin (O, P) followed by mock treatment (M, O) or ISO treatment (N, P). β2-AR, beta-2 adrenergic receptor; EGF, epidermal growth factor; EGFR, epidermal growth factor receptor; ISO, isoproterenol; WT, wild type.
(TIF)

S11 Fig Measurement of an equilibrium dissociation constant of the interaction of SNAP-EGFR with PMT-mEos3.2, EGFR-mEos3.2, and mEos3.2-EGFR. (A) Using Co-II, the K_D_ of interaction between SNAP-EGFR and PMT-mEos3.2 was measured as 26,890 ± 2,724 molecules/μm^2^ and compared with the K_D_ of EGFR pre-homodimerization in DMEM supplemented with 10% FBS at 37 °C. (B) The K_D_ of the interaction of SNAP-EGFR with EGFR-mEos3.2 and mEos3.2-EGFR. The K_D_ of EGFR pre-homodimerization was not affected by the orientation of mEos3.2. The error bars represent the SEM at the single-cell level (*n* > 10). DMEM, Dulbecco's Modified Eagle Medium; EGFR, epidermal growth factor receptor; FBS, fetal bovine serum; mEos3.2, monomeric Eos fluorescent protein variant 3.2; n.s., nonsignificant difference. PMT, plasma membrane targeting; SNAP, SNAP-tag.
(TIF)

S12 Fig The estimation of relative EGFR oligomer size using Co-II. The frequency of stopping single-molecule EGFR prey in the vicinity of the immobilized one with the EGFR trajectories collected for 5 min in the absence (A) and presence (B) of EGF was analyzed using the neighbor search algorithm with a distance of 20 nm. To minimize the overcounting issue derived from the blinking of mEos3.2, we counted the stopping mEos3.2 with a blinking tolerance time of 4 s, as previously described [55], which might result in the loss of weak oligomerization. The relative oligomer size distribution of EGFR interactions was obtained by subtracting the distribution of stopping EGFR after the bait immobilization by the distribution before the immobilization. EGF, epidermal growth factor; EGFR, epidermal growth factor receptor; mEos3.2, monomeric Eos fluorescent protein variant 3.2.
(TIF)

S13 Fig Theoretical uncertainty of K_D_ determination in a single cell using Co-II. Relative K_D_ uncertainty with respect to a co-immobilized fraction and the uncertainty of co-immobilized fraction, ε. The uncertainty in K_D_ increased exponentially in the range near both boundaries of the co-immobilized fraction. Optimization of the expression level of a bait protein is crucial to achieve minimal uncertainty of K_D_ in a single cell because the co-immobilized fractions depend on the expression level of the bait with a given K_D_, according to Eq 3 in Materials and methods. The red line indicates the expected experimental uncertainty in this study.
(TIF)

## Abbreviations

β2-AR: beta-2 adrenergic receptor
BRET: bioluminescence resonance energy transfer
cryo-EM: cryo-electron microscopy
DMEM: Dulbecco's Modified Eagle Medium
ECD: extracellular domain
EGF: epidermal growth factor
EGFR: epidermal growth factor receptor
ErbB2: erb-b2 receptor tyrosine kinase 2
ErbB3: erb-b2 receptor tyrosine kinase 3
Fab: fragment antigen-binding
FBS: fetal bovine serum
FRET: fluorescence resonance energy transfer
ICD: intracellular domain
mEos3.2: monomeric Eos fluorescent protein variant 3.2
MSD: mean squared displacement
PMT: plasma membrane targeting
SPT: single-particle tracking
sptPALM: single-particle tracking photoactivated localization microscopy
TIRF: total internal reflection fluorescence
Y2H: yeast two-hybrid
