# Supplementary figures and images for: Direct visualization of single-molecule membrane protein interactions in living cells
Source: PLoS Biol. 2018 Dec 13;16(12):e2006660. doi: 10.1371/journal.pbio.2006660 (PMC6307816; doi:10.1371/journal.pbio.2006660)

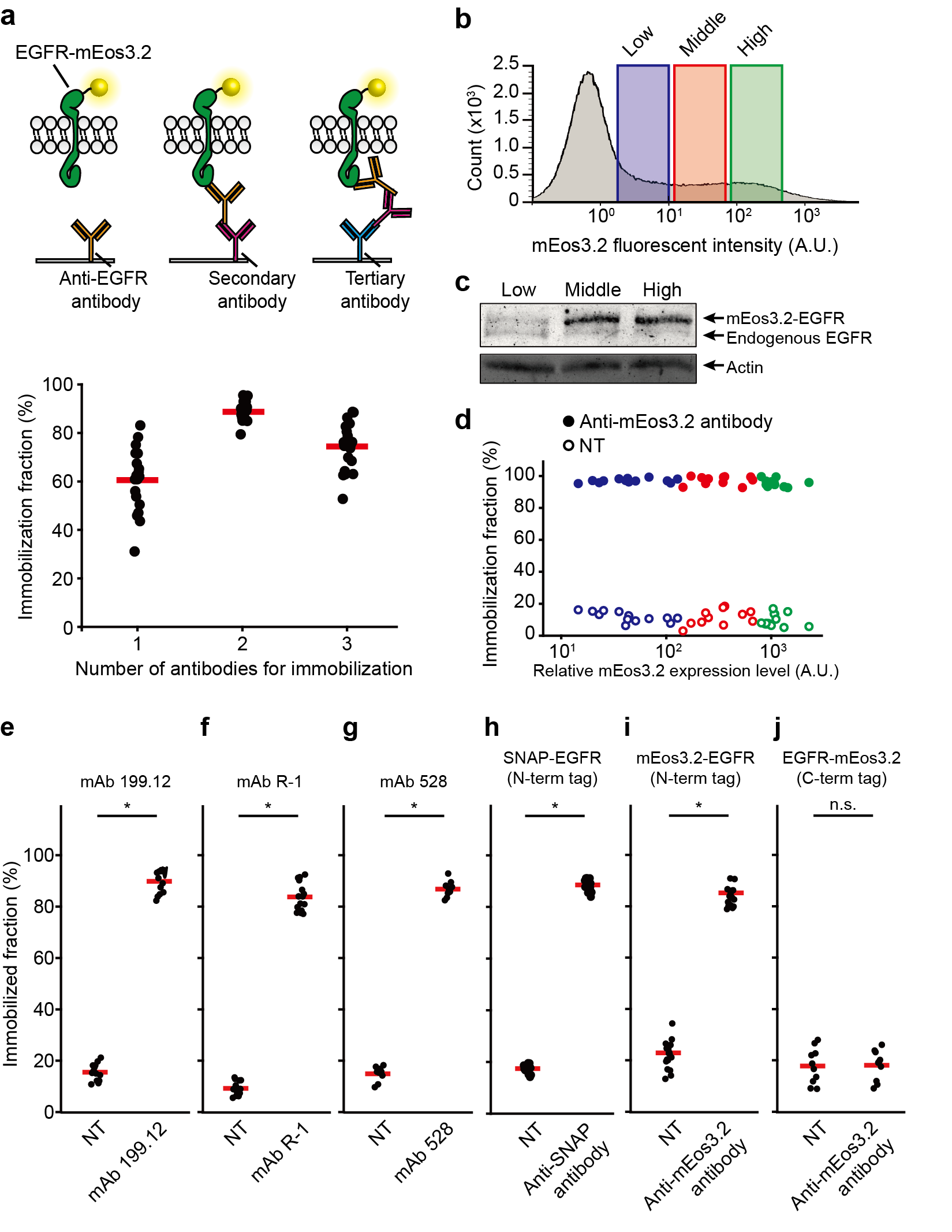

Supplement: S1 Fig — (A) Illustration and immobilization efficiency of the three types of immobilization systems for EGFR-mEos3.2 of varying heights from the glass surface in COS7 cells. Each dot represents single-cell data, and the red solid lines indicate the average of the immobilized fractions obtained from multiple cells (n > 10). (B) COS7 cells expressing mEos3.2-EGFR were sorted into three parts based on the expression level (low, middle, and high) using the mEos3.2 fluorescence intensity. (C) The immobilized fractions of mEos3.2-EGFR before (empty circle) and after mEos3.2 antibody treatment (filled circle) were constant across all three levels of EGFR expression (low: blue circles, middle: red circles, high: green circles), as calculated by the mEos3.2 fluorescence intensity from TIRF images. Each dot represents single-cell data. (D–F) The immobilized fraction of EGFR before and after treatment with three different anti-EGFR antibody clones with different EGFR binding epitopes: mAb 199.12 (D), mAb R-1 (E), and mAb 528 (F). (G) The immobilized fraction of EGFR before and after anti-SNAP antibody treatment in cells expressing SNAP-EGFR. (H–I) The immobilized fraction of EGFR before and after treatment with an anti-mEos3.2 antibody in cells expressing mEos3.2-EGFR (H) and EGFR-mEos3.2 (I). Each dot represents single-cell data, and the red solid lines indicate the average of the immobilized fractions obtained from multiple cells (n > 10). *p < 0.05 (Student t test). EGFR, epidermal growth factor receptor; mEos3.2, monomeric Eos fluorescent protein variant 3.2; n.s., nonsignificant difference; SNAP, SNAP-tag; TIRF, total internal reflection fluorescence. (TIF) [file pbio.2006660.s002.tif]

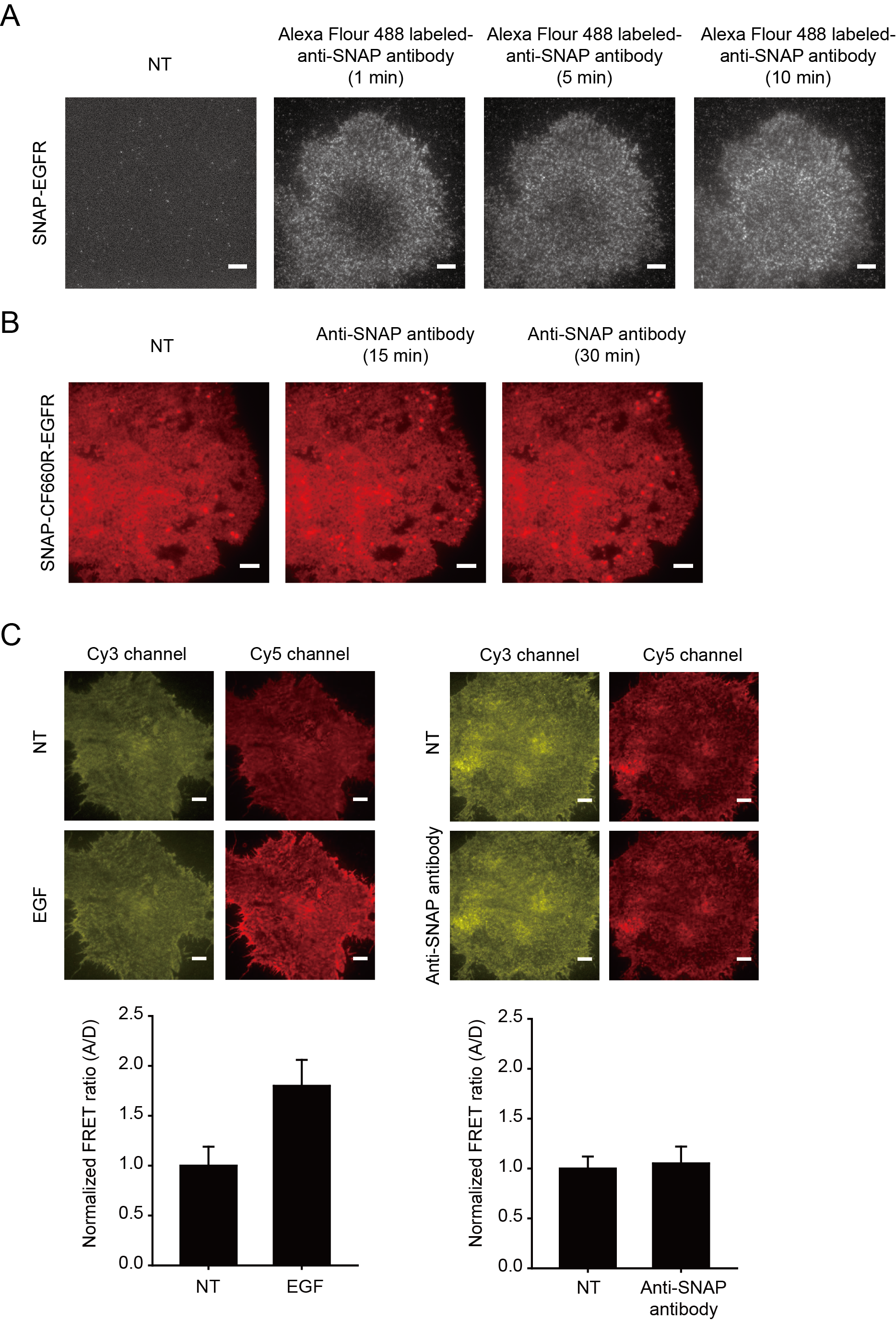

Supplement: S2 Fig — (A) Alexa Fluor 488–labeled anti-SNAP antibody was treated to a COS7 cell expressing SNAP-EGFR (non-labeled) seeded on a cleaned glass to visualize the process of the antibody penetration between the cell bottom and the glass surface. The antibody was fully penetrated across the entire cell surface within 10 min. (B) The anti-SNAP antibody was treated to a COS7 cell expressing SNAP-EGFR labeled by BG-CF660R seeded on the anti-rabbit secondary antibody-coated glass to observe the effect of the antibody-induced SNAP-EGFR immobilization on the distribution of EGFR on the plasma membrane. No significant change in EGFR distribution on the plasma membrane was detected. (C) FRET experiments were performed to examine whether the cross-linking of SNAP-EGFR is produced by the surface immobilization using anti-SNAP antibody. BG-Cy3 and BG-Cy5 were treated at 1:1 ratio on COS7 cells expressing SNAP-EGFR seeded on the anti-rabbit secondary antibody-coated glass. Both Cy3 (donor) and Cy5 (acceptor) channels were monitored with a donor-only excitation. Then, the cells were treated with EGF or anti-SNAP antibody. FRET ratios (acceptor/donor) were normalized to analyze the relative changes in FRET ratios by the treatments (n > 5). No significant cross-linking was observed by the anti-SNAP antibody induced SNAP-EGFR immobilization. Scale bars, 5 μm. BG, benzyl guanine; EGF, epidermal growth factor; EGFR, epidermal growth factor receptor; FRET, fluorescence resonance energy transfer; SNAP, SNAP-tag. (TIF) [file pbio.2006660.s003.tif]

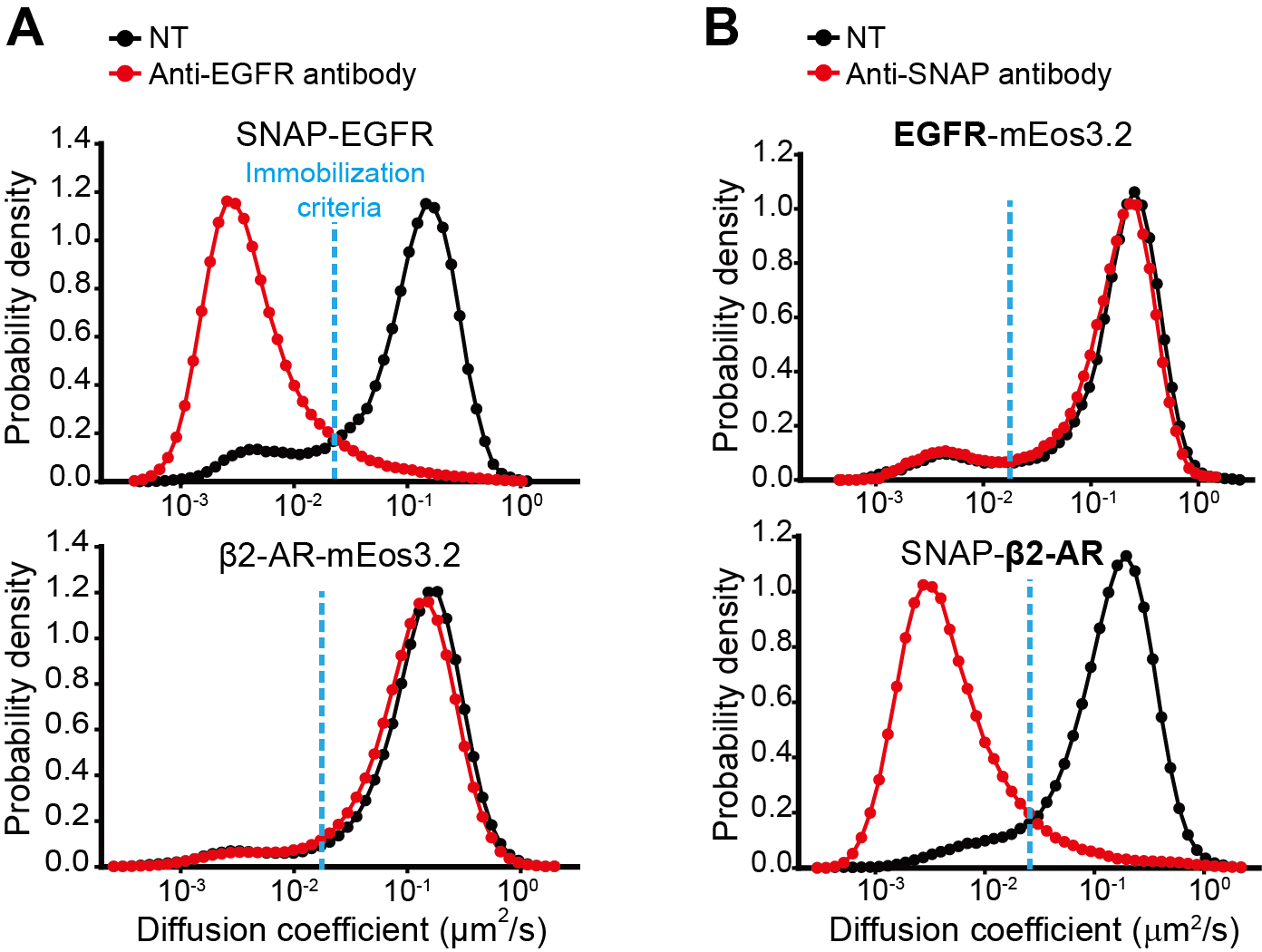

Supplement: S3 Fig — (A) Diffusion-coefficient distributions of SNAP-EGFR and β2-AR-mEos3.2 before (black lines) and after anti-EGFR antibody treatment (red lines). (B) Diffusion-coefficient distributions of EGFR-mEos3.2 and SNAP-β2-AR before (black lines) and after anti-SNAP antibody treatment (red lines). β2-AR, beta-2 adrenergic receptor; EGFR, epidermal growth factor receptor; mEos3.2, monomeric Eos fluorescent protein variant 3.2; SNAP, SNAP-tag. (TIF) [file pbio.2006660.s004.tif]

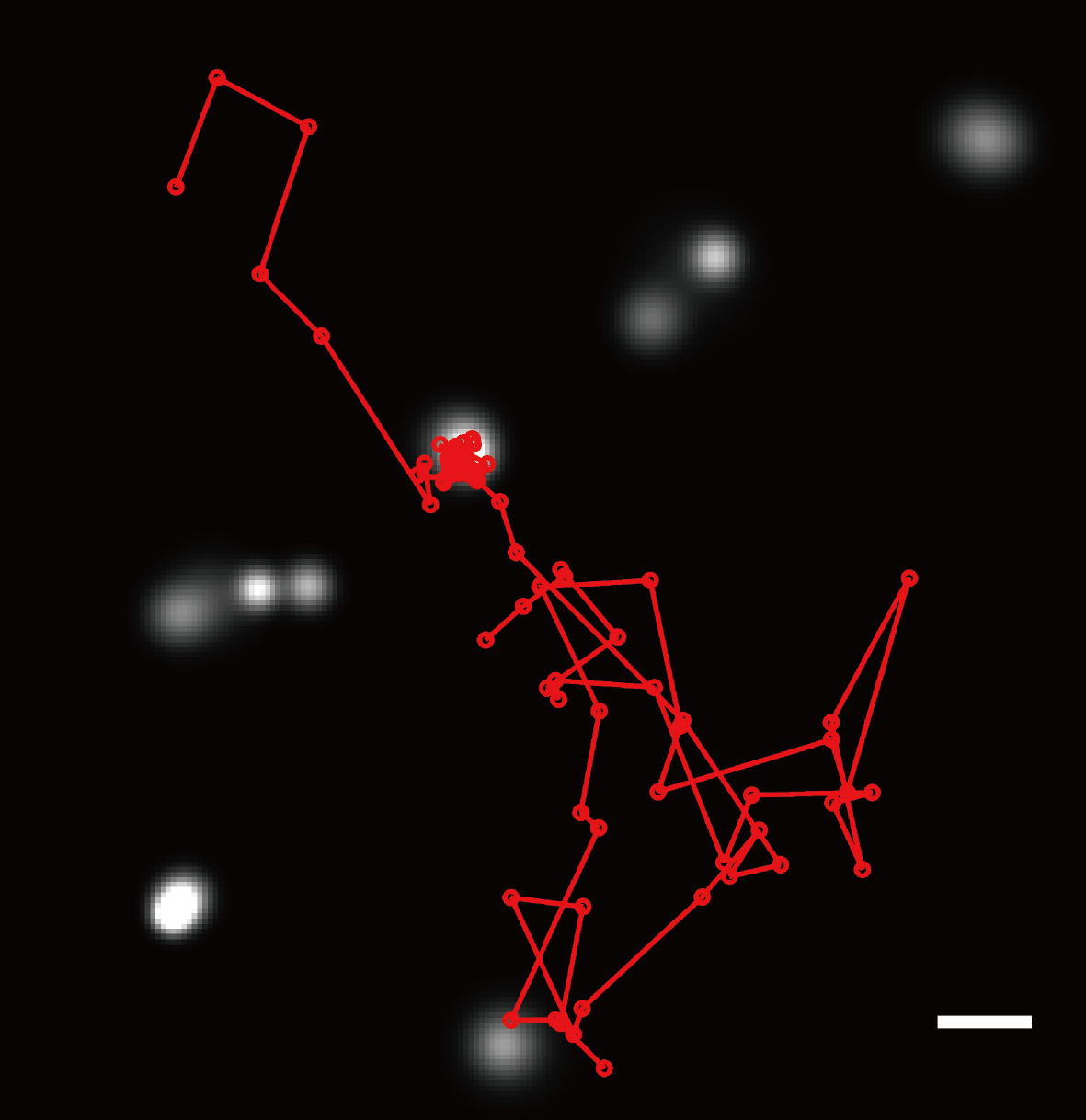

Supplement: S4 Fig — The red line indicates a single molecule trajectory of SNAP-EGFR labeled with Alexa Fluor 647 (the prey), and the white dots represent antibody-induced immobilized mEos3.2-EGFR (the bait). To acquire long trajectories to observe the transition of mobile-immobile-mobile states, we utilized benzyl-guanine–conjugated Alexa Fluor 647 instead of mEos3.2. Therefore, we immobilized mEos3.2 using anti-mEos3.2 antibody instead of the SNAP tag. The temporarily immobilized SNAP-EGFR was colocalized with the antibody-induced immobilized mEos3.2-EGFR within 30 nm. Scale bar, 500 nm. EGFR, epidermal growth factor receptor; mEos3.2, monomeric Eos fluorescent protein variant 3.2; SNAP, SNAP-tag. (TIF) [file pbio.2006660.s005.tif]

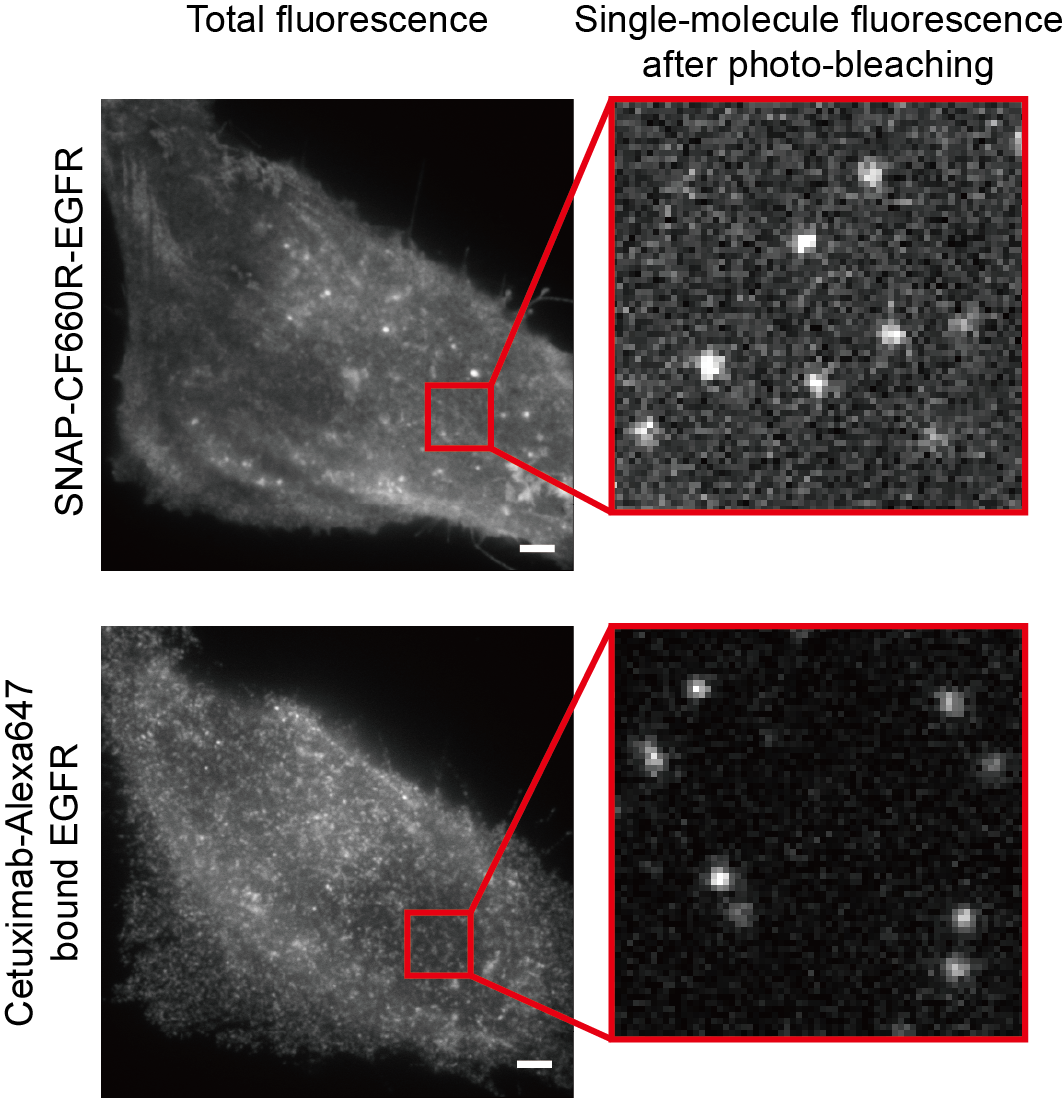

Supplement: S5 Fig — The fluorescent SNAP-CF660R-EGFR ratio was determined. TIRF image of the total expression and single-molecule fluorescence of SNAP-CF660R-EGFR and cetuximab-Alexa Fluor 647–labeled EGFR in HeLa cells, which marginally express endogenous EGFR. Scale bar, 5 μm. The ratio between protein concentrations quantified using CF660R-SNAP and cetuximab-Alexa Fluor 647 was 0.91 ± 0.13. EGFR, epidermal growth factor receptor; SNAP, SNAP-tag; TIRF, total internal reflection fluorescence. (TIF) [file pbio.2006660.s006.tif]

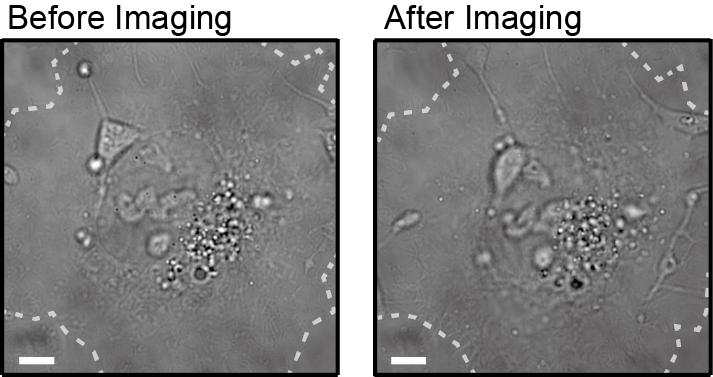

Supplement: S6 Fig — DIC images were taken before and after performing the Co-II assay in the same cell. Photodamage to cell morphology was undetectable. Scale bar, 5 μm. DIC, differential interference contrast. (TIF) [file pbio.2006660.s007.tif]

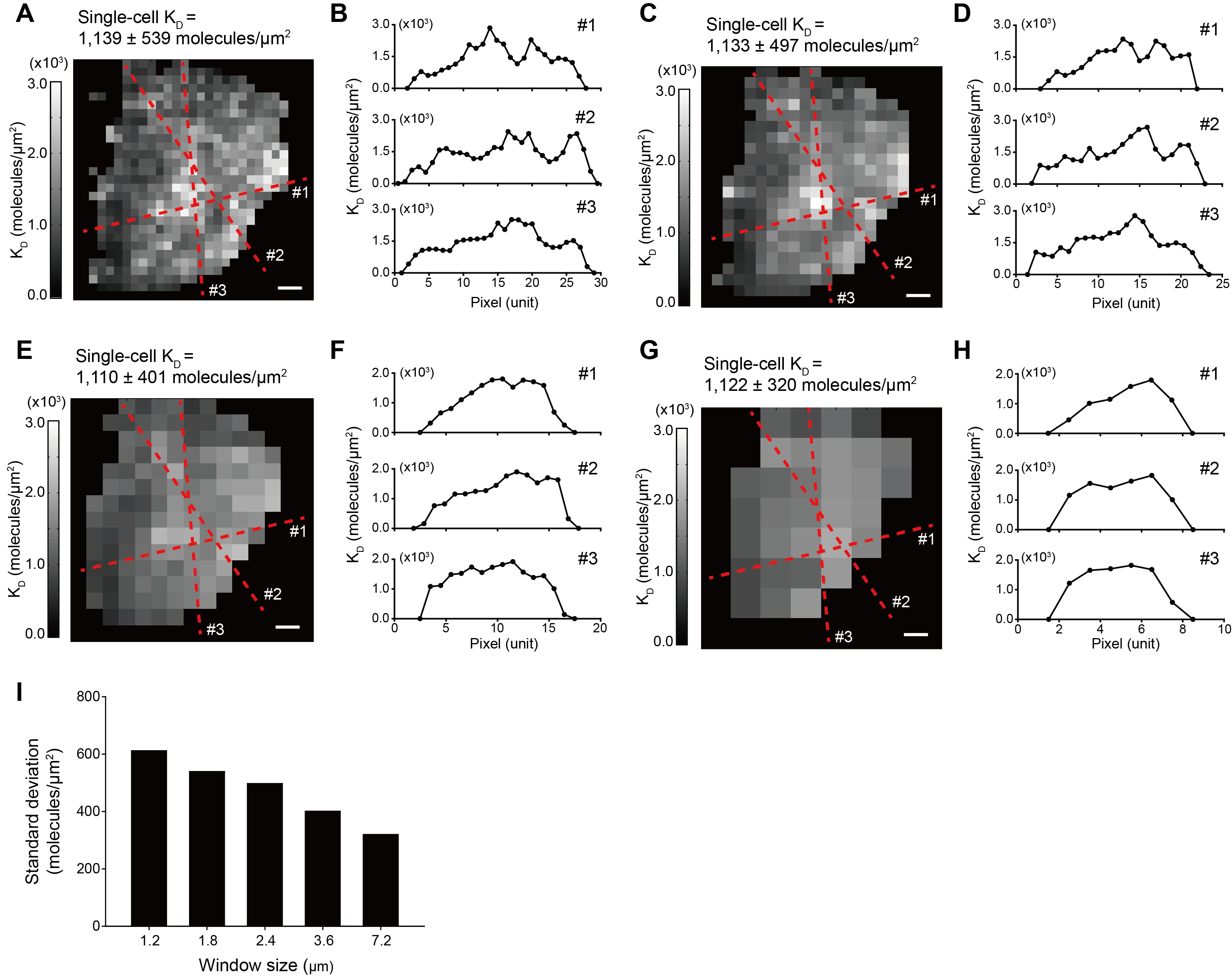

Supplement: S7 Fig — (A, C, E, G) Spatial KD maps of EGFR pre-homodimerization in a single living cell with different sizes of average window (1.2 μm, 1.8 μm, 2.4 μm, and 3.6 μm, repectively). Scale bar, 5 μm. (B, D, F, H) The KD profiles obtained from cross sections corresponding to the red dashed lines in respective panels. (I) Standard deviation of KD maps with different sizes of average window. EGFR, epidermal growth factor receptor. (TIF) [file pbio.2006660.s008.tif]

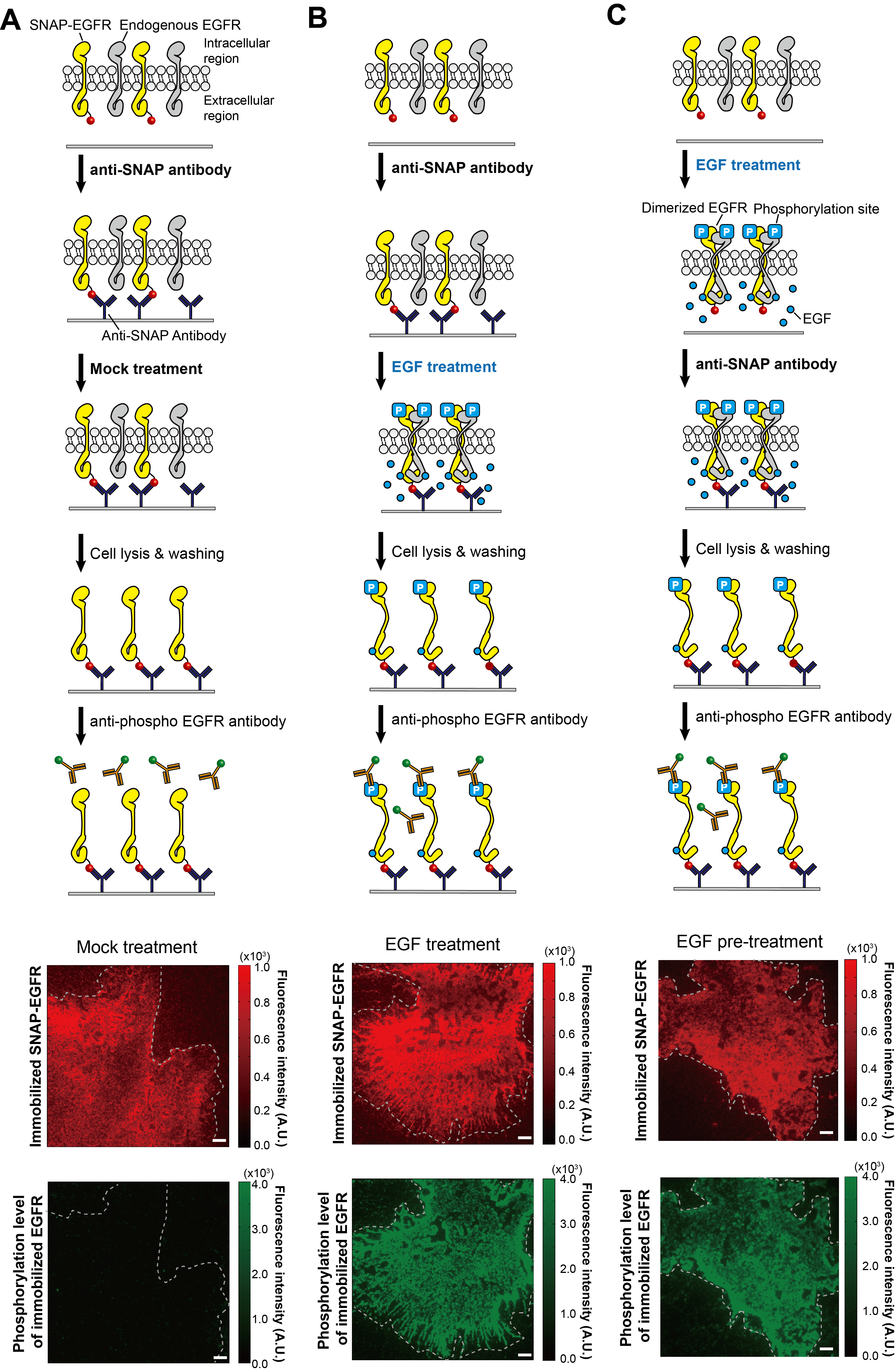

Supplement: S8 Fig — SNAP-EGFR expressed in COS7 cells was immobilized onto a glass surface using an anti-SNAP antibody. The cells were lysed after mock (A) or EGF (B) treatment, resulting in SNAP-EGFR being held by the antibody coated on a glass surface. As a positive control, the cells were treated by EGF first, then immobilized onto a glass surface (C). The phosphorylation level of the remaining SNAP-EGFRs was measured using an anti-EGFR Y1068 antibody and an Alexa Fluor 488–conjugated secondary antibody. Cells with similar levels of SNAP-EGFR expression were examined to compare the phosphorylation of the remaining SNAP-EGFRs with or without EGF treatment. Scale bar, 5 μm. An enormously elevated level of phosphorylation induced by EGF was detected compared with the basal level of phosphorylation without EGF treatment, whereas a similar level of phosphorylation was observed regardless of the order of the EGF treatment and the SNAP-EGFR immobilization. EGF, epidermal growth factor; EGFR, epidermal growth factor receptor; SNAP, SNAP-tag. (TIF) [file pbio.2006660.s009.tif]

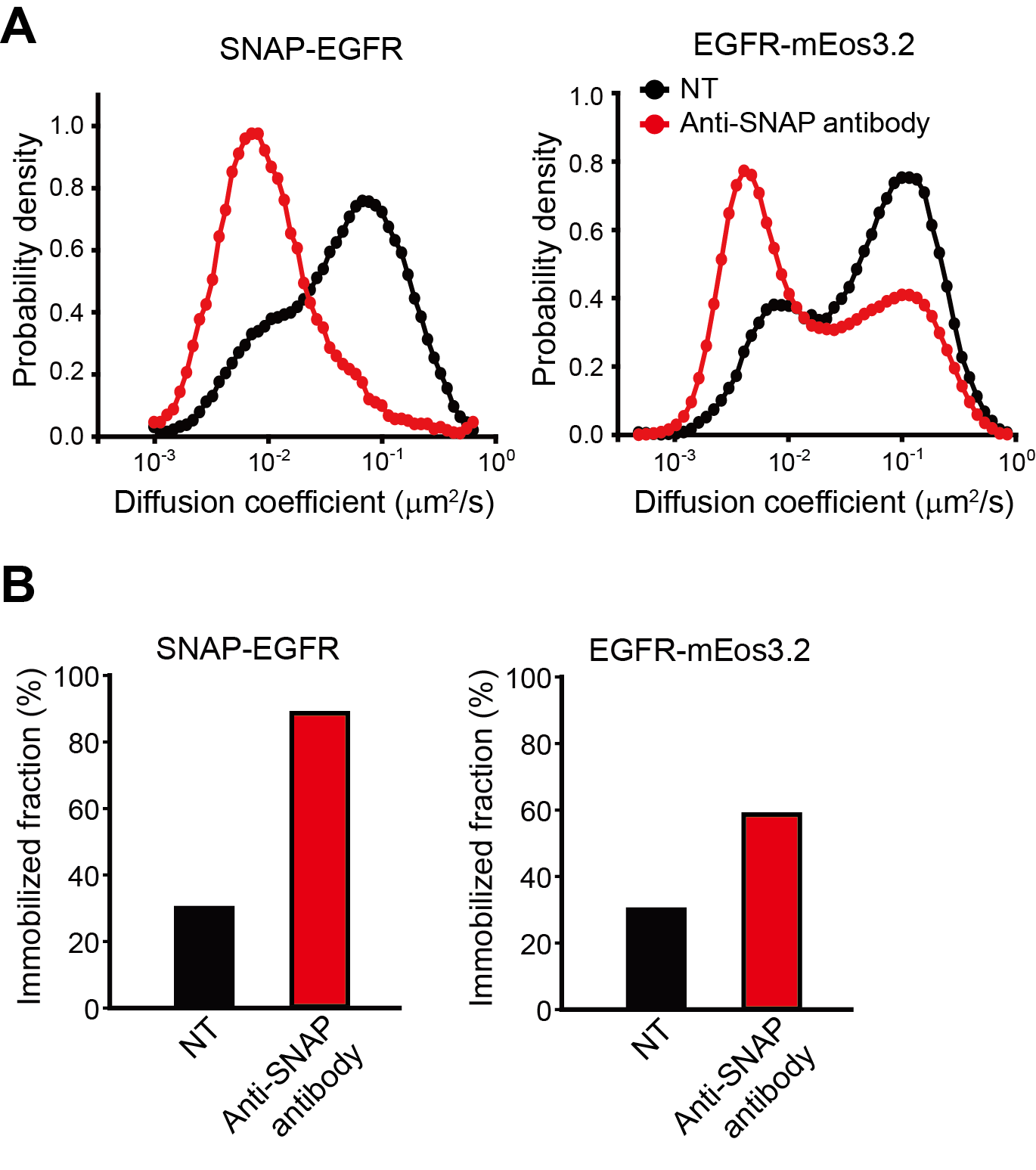

Supplement: S9 Fig — (A) Diffusion-coefficient distribution of SNAP-EGFR and EGFR-mEos3.2 before (black line) and after anti-SNAP antibody treatment (red line) in a EGF-treated COS7 cell. (B) The immobilized fractions of SNAP-EGFR and EGFR-mEos3.2 before and after anti-SNAP antibody treatment. EGF, epidermal growth factor; EGFR, epidermal growth factor receptor; mEos3.2, monomeric Eos fluorescent protein variant 3.2; SNAP, SNAP-tag. (TIF) [file pbio.2006660.s010.tif]

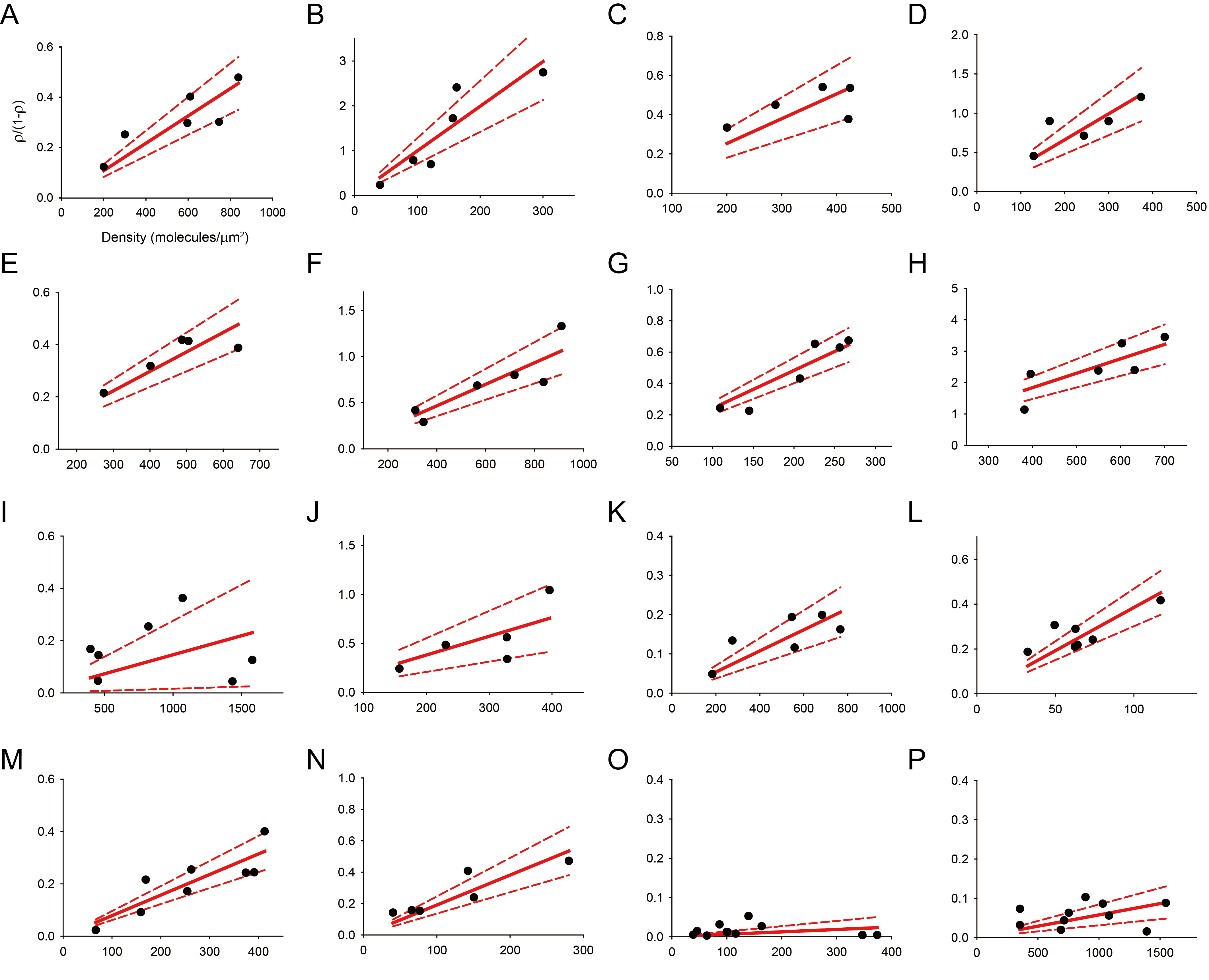

Supplement: S10 Fig — Co-immobilized fractions, ρ, determined from Co-II assay were analyzed from the binding curves to estimate KD values (n ≥ 5). Because KD = (1−ρ)/ρ*[I]0 in Co-II, we utilized a linear fit to reduce the complexity of the curve fitting using the equation, ρ/(1−ρ) = a*[I]0, where a is the slope of the linear fit (a solid red line) representing 1/KD, and [I]0 is the density of antibody-induced immobilized bait proteins. The 95% confidence intervals are shown (dashed red lines). (A–J) The analysis of the homodimerization of EGFR WT pretreated with mock (A, B), erlotinib (C, D), lapatinib (E, F), nystatin (G, H), and cetuximab (I, J) followed by mock treatment (A, C, E, G, I) or EGF treatment (B, D, F, H, J). (K, L) The analysis of the homodimerization of EGFR L858R (K) or EGFRvIII (L). (M–P) The analysis of the homodimerization of β2-AR WT pretreated with mock (M, N) or nystatin (O, P) followed by mock treatment (M, O) or ISO treatment (N, P). β2-AR, beta-2 adrenergic receptor; EGF, epidermal growth factor; EGFR, epidermal growth factor receptor; ISO, isoproterenol; WT, wild type. (TIF) [file pbio.2006660.s011.tif]

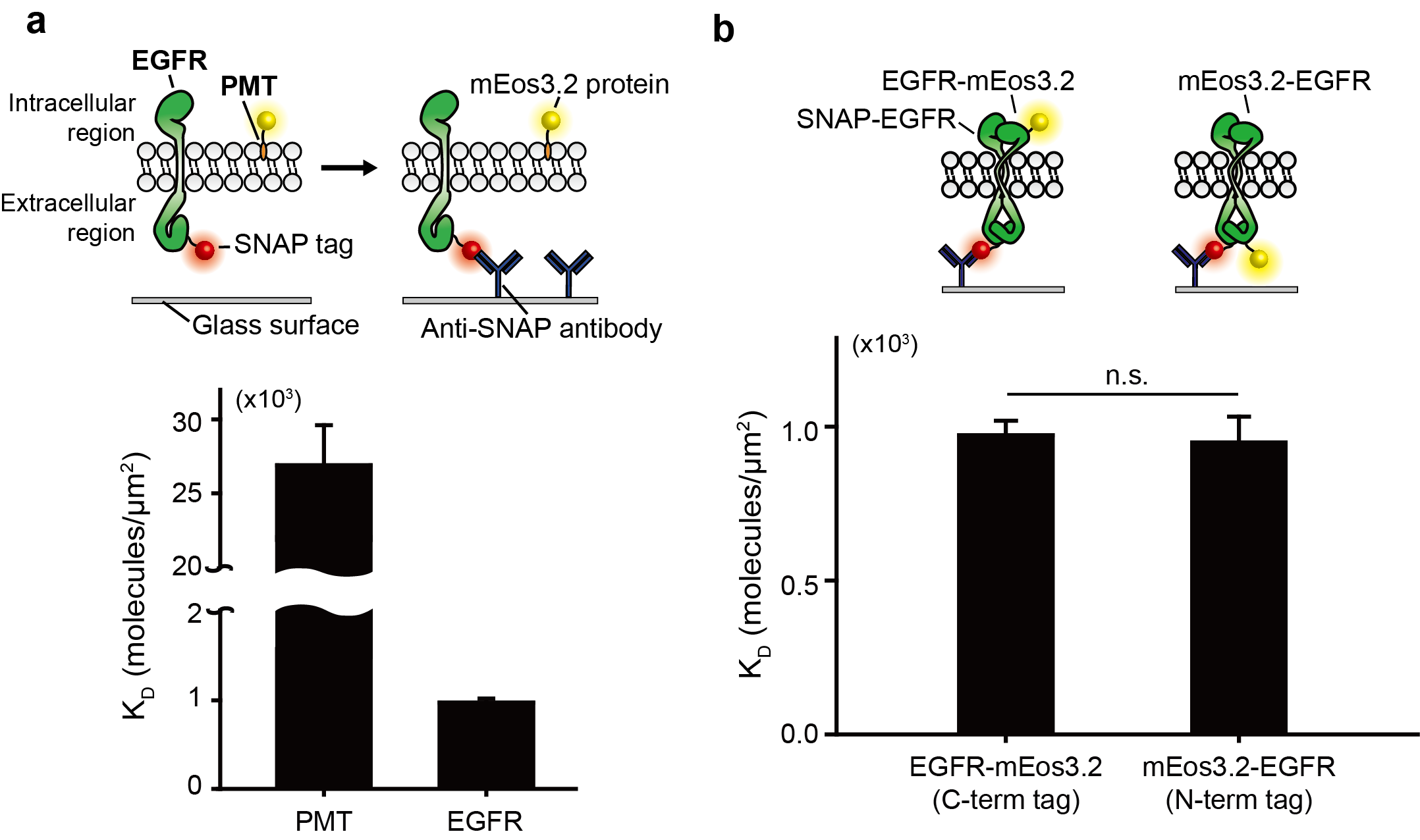

Supplement: S11 Fig — (A) Using Co-II, the KD of interaction between SNAP-EGFR and PMT-mEos3.2 was measured as 26,890 ± 2,724 molecules/μm2 and compared with the KD of EGFR pre-homodimerization in DMEM supplemented with 10% FBS at 37 °C. (B) The KD of the interaction of SNAP-EGFR with EGFR-mEos3.2 and mEos3.2-EGFR. The KD of EGFR pre-homodimerization was not affected by the orientation of mEos3.2. The error bars represent the SEM at the single-cell level (n > 10). DMEM, Dulbecco's Modified Eagle Medium; EGFR, epidermal growth factor receptor; FBS, fetal bovine serum; mEos3.2, monomeric Eos fluorescent protein variant 3.2; n.s., nonsignificant difference. PMT, plasma membrane targeting; SNAP, SNAP-tag. (TIF) [file pbio.2006660.s012.tif]

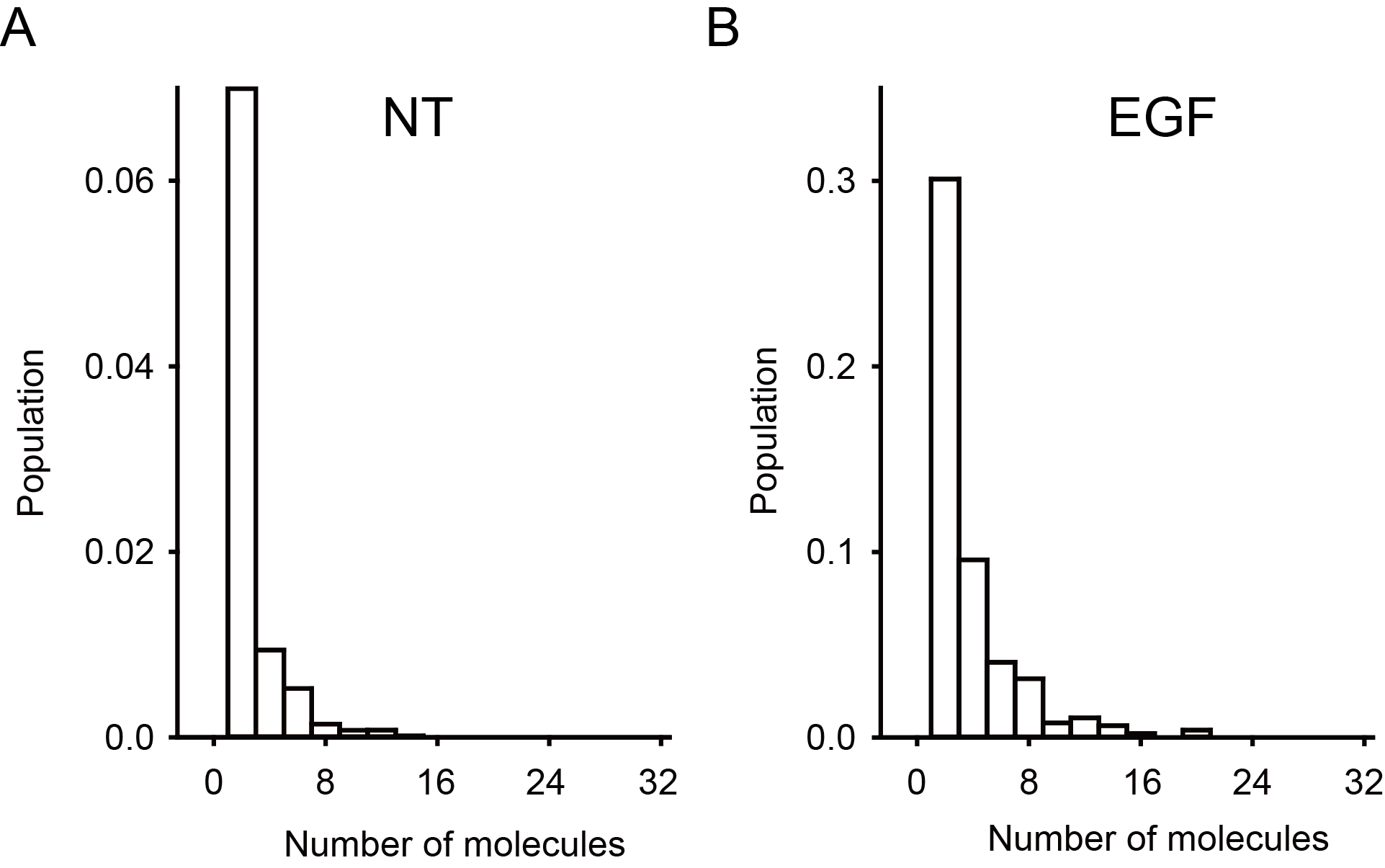

Supplement: S12 Fig — The frequency of stopping single-molecule EGFR prey in the vicinity of the immobilized one with the EGFR trajectories collected for 5 min in the absence (A) and presence (B) of EGF was analyzed using the neighbor search algorithm with a distance of 20 nm. To minimize the overcounting issue derived from the blinking of mEos3.2, we counted the stopping mEos3.2 with a blinking tolerance time of 4 s, as previously described [55], which might result in the loss of weak oligomerization. The relative oligomer size distribution of EGFR interactions was obtained by subtracting the distribution of stopping EGFR after the bait immobilization by the distribution before the immobilization. EGF, epidermal growth factor; EGFR, epidermal growth factor receptor; mEos3.2, monomeric Eos fluorescent protein variant 3.2. (TIF) [file pbio.2006660.s013.tif]

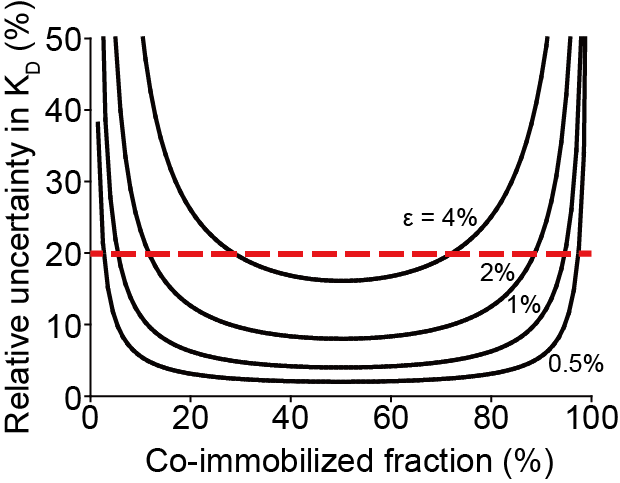

Supplement: S13 Fig — Relative KD uncertainty with respect to a co-immobilized fraction and the uncertainty of co-immobilized fraction, ε. The uncertainty in KD increased exponentially in the range near both boundaries of the co-immobilized fraction. Optimization of the expression level of a bait protein is crucial to achieve minimal uncertainty of KD in a single cell because the co-immobilized fractions depend on the expression level of the bait with a given KD, according to Eq 3 in Materials and methods. The red line indicates the expected experimental uncertainty in this study. (TIF) [file pbio.2006660.s014.tif]
